# *Parkia javanica* Edible Pods Reveal Potential as an Anti-Diabetic Agent: UHPLC-QTOF-MS/MS-Based Chemical Profiling, In Silico, In Vitro, In Vivo, and Oxidative Stress Studies

**DOI:** 10.3390/ph17070968

**Published:** 2024-07-21

**Authors:** Alekhya Sarkar, Arjita Chakrabarti, Samhita Bhaumik, Bimal Debnath, Shiv Shankar Singh, Rajat Ghosh, Magdi E. A. Zaki, Sami A. Al-Hussain, Sudhan Debnath

**Affiliations:** 1 Department of Forestry and Biodiversity, Tripura University, Suryamaninagar 799022, India; alekhya.sarkar@gmail.com (A.S.); bimaldebnath@tripurauniv.ac.in (B.D.); 2 Department of Zoology, Tripura University, Suryamaninagar 799022, India; arjitachakrabarti95@gmail.com (A.C.); shivssingh@tripurauniv.ac.in (S.S.S.); 3 Department of Chemistry, Women’s College, Agartala 799001, India; samhitabhaumik@gmail.com; 4 In Silico Drug Design Lab., Department of Pharmacy, Tripura University, Suryamaninagar 799022, India; rajatghosh@tripurauniv.ac.in; 5 Department of Chemistry, Faculty of Science, Imam Mohammad Ibn Saud Islamic University, Riyadh 11623, Saudi Arabia; sahussain@imamu.edu.sa; 6 Department of Chemistry, Netaji Subhash Mahavidyalaya, Udaipur 799114, India

**Keywords:** *Parkia javanica*, UHPLC-QTOF-MS/MS, phytochemicals, molecular docking, α-glucosidase, in vitro, in vivo, diabetes, oxidative stress

## Abstract

According to the World Health Organization, over 422 million people worldwide have diabetes, with the majority residing in low- and middle-income countries. Diabetes causes 1.5 million fatalities a year. The number of diabetes cases and its prevalence have progressively increased over the last few decades. This study aims to determine the phytochemicals in the edible part of *Perkia javanica*, predict their α-glucosidase inhibitory potential, one of the promising targets for diabetes, and then carry out in vitro and in vivo studies. The phytochemicals present in the n-butanol fraction of the methanol extract of *P*. *javanica* pods were analyzed using UHPLC-QTOF-MS/MS (Ultra-High-Performance Liquid Chromatography-Quadrupole Time-of-Flight Mass Spectrometry). The UHPLC-QTOF analysis revealed the presence of 79 different compounds in the n-butanol fraction. Among these, six compounds demonstrated excellent binding affinities with α-glucosidase, surpassing the performance of two standard inhibitors, Miglitol and Voglibose. In vitro α-glucosidase inhibitory activities were assessed by the n-butanol fraction, followed by in vivo studies. According to the in vitro study, the inhibitory efficiency against α-glucosidase was determined to have an IC_50_ value of 261.9 µg/mL. The in vivo findings revealed a significant reduction in blood glucose levels in Swiss albino mice treated with the same extract, decreasing from 462.66 mg/dL to 228.66 mg/dL. Additionally, the extract significantly increased the activity of the enzymes catalase and superoxide dismutase (SOD) and decreased the amount of malondialdehyde (MDA) in the liver and kidney tissue. The predicted physicochemical parameters indicated that most of the compounds would be excreted from the body after inhibition in the small intestine without being absorbed. Considering the low cost and wide availability of raw materials, *P. javanica* pods can serve as a good food supplement that may help prevent type 2 diabetes management.

## 1. Introduction

Just about 500 million people worldwide have diabetes at the moment, but by 2030 and 2045, that number is predicted to increase to 51% and 25%, respectively [1]. In 2003, the anticipated yearly cost of diabetes treatment in India was between Rs. 10,000 and Rs. 12,000 crore. By 2025, this amount is expected to reach up to Rs. 126,000 crore [2]. Diabetes has numerous financial repercussions, both directly and indirectly, around the globe, and these effects impose a substantial burden on society. The two main types of diabetes mellitus are type 1 and type 2. Type 2 diabetes comprises more than 95% of people with diabetes. Type 2 diabetes is referred to as adult-onset or non-insulin-dependent diabetes. Major classes of drugs used in the treatment of DM are insulins, sulfonylureas (SUs), thiazolidinediones (TZDs), biguanides, meglitinides, α-glucosidase inhibitors (AGIs), dipeptidyl peptidase-4 (DPP-4) inhibitors, glucagon-like peptide-1 (GLP-1) agonists, sodium-glucose cotransporter 2 (SGLT2) inhibitors, and dopamine agonists [3]. One effective treatment approach for lowering blood glucose associated with type 2 diabetes is to inhibit α-amylase, α-glucosidase, or both, the enzymes that catalyze the intestinal hydrolysis of starch. In the last stage of glucose digestion, α-glucosidase catalyzes the hydrolysis of the α-(1,4)-glycosidic bond of sugar, releasing free monosaccharide (α-D-glucose) [4]. α-Glucosidase enzymes, in particular, are inhibited by α-glucosidase inhibitors in the small intestine. The recognized α-glucosidase inhibitors at the moment are Voglibose, Miglitol, and Acarbose [5,6]. Current long-term anti-diabetes medications have several side effects, which emphasizes the significance of adopting natural products. Medicinal plants commonly used for the treatment and management of diabetes in different systems of medicine like Traditional Chinese Medicine (TCM), Traditional Japanese Medicine (Kampo), Traditional Indian Medicine (Ayurveda), European Herbal Medicine, and Russian Traditional Medicine [7,8,9,10]. Recent research has identified anti-diabetes compounds sourced from plants using computational approaches [11,12,13,14]. This study focuses on finding α-glucosidase inhibitors or a mixture of compounds as inhibitors from natural sources. Thus, efforts to find new inhibitors with better efficacy and reduced side effects are still ongoing. It has been suggested that more than 1200 blooming plants have anti-diabetic potential. Among them, one-third were scientifically validated and published in around 460 articles [15]. Some plants exhibit hypoglycemic, anti-hyperglycemic, and anti-diabetic properties with α-amylase and α-glucosidase inhibitory potential [16,17]. The indigenous inhabitants of the eastern Himalayan region of India are known to use herbal remedies either on their own or in conjunction with other types of treatment to cure diabetes [18]. Therefore, natural products may be safer than synthetic medications in treating diabetes [19]. α-Glucosidase inhibitors, such as Acarbose, Miglitol, and Voglibose, have been used clinically since 1990 to manage type 2 diabetes by slowing down the digestion and absorption of carbohydrates. Despite being useful in regulating blood glucose levels, they are often associated with gastrointestinal side effects [20,21]. Therefore, finding new α-glucosidase inhibitors or edible herbal products with fewer side effects is urgently needed. Target-based in silico analysis, combined with modern chemical profiling methods, saves both money and time [12,22,23]. Numerous research teams have identified several plant-based anti-diabetic agents [24,25,26,27]. The aforementioned results motivate the current researcher to use cutting-edge methods to determine the potential of edible pods of *P. javanica* to prevent diabetes. *Parkia javanica* (local name: Kuki Tetai, Youngchak) is a medium-sized tree of the Mimosaceae family that grows abundantly in the northeastern states of India. Tender pods are mostly used as vegetables [28]. Various parts of this plant are used in traditional medicine [29]. It was stated that 49 *Parkia timoriana* compounds were identified by GC-MS, revealing their anti-inflammatory and anti-cancer properties [30]. An investigation using pod extract revealed its anti-biofilm activity against *Pseudomonas aeruginosa*. The pods have been used traditionally to treat diabetes, kidney pain, and cholera [31]. The *P. javanica* pods have the ability to induce apoptosis in cancer cells, and the methanolic extract of bark exhibits cytotoxic potential against a colon cancer cell line [32,33,34]. Studies showed that *P. javanica pods* have high antioxidant properties [35] and in vitro activity against the *Leishmania donovani* parasite [36,37]. *P*. *javanica* pod extract also possesses potent anti-angiogenic and antiproliferative properties [38]. The major compounds reported so far from *P. javanica* are 2,4-Di-tert-butylphenol, baicalein, quercetin, chrysin, cinnamic acid, tannic acid, resorcinol, 8-*O*-p-hydroxybenzoyl-6′-*O*-p-coumaroyl-mussaenosidic acid, and 7-*O*-E-3,4-dimethoxycinnamoyl-6′-*O*-b-d-glucopyranosylloganic acid [31,39,40,41]. The total number of compounds reported so far is inadequate. Exploring more compounds from this important medicinal plant is urgently needed, which may open up new avenues in the field of anti-cancer and anti-diabetes drug discovery. As of now, no study has been conducted to investigate the UHPLC-QTOF-MS/MS-based chemical profiling of compounds of *P. javanica* and their anti-diabetic potential. In the present study, UHPLC-QTOF-MS/MS-based chemical profiling, in vitro α-glucosidase inhibitory potential, in vivo anti-diabetic activity, and oxidative stress of the n-butanol fraction of the methanol extract of *P. javanica* pods were investigated.

## 2. Results

### 2.1. Chemical Profiling

The n-butanol fraction of the methanol extract of *P*. *javanica* was used for UHPLC-QTOF-MS/MS-based analysis. The positive and negative ionization modes were employed, and a series of 56 (PJ_01–PJ_56) and 23 (PJ_57–PJ_79) compounds were identified, respectively. The spectra of both positive and negative ionization modes are shown in Figures S1 and S2, respectively. The separated compounds were analyzed within the *m*/*z* 100–1500 mass range with increasing retention time and identified by comparing the METLIN Database [42]. All the compounds detected by UPLC-MS through the positive and negative modes are shown in Table 1 and Table 2, respectively. The structures of all the compounds are shown in Figure S3.

### 2.2. In Silico Studies of Phytochemicals

#### Molecular Docking

**Figure 1:**
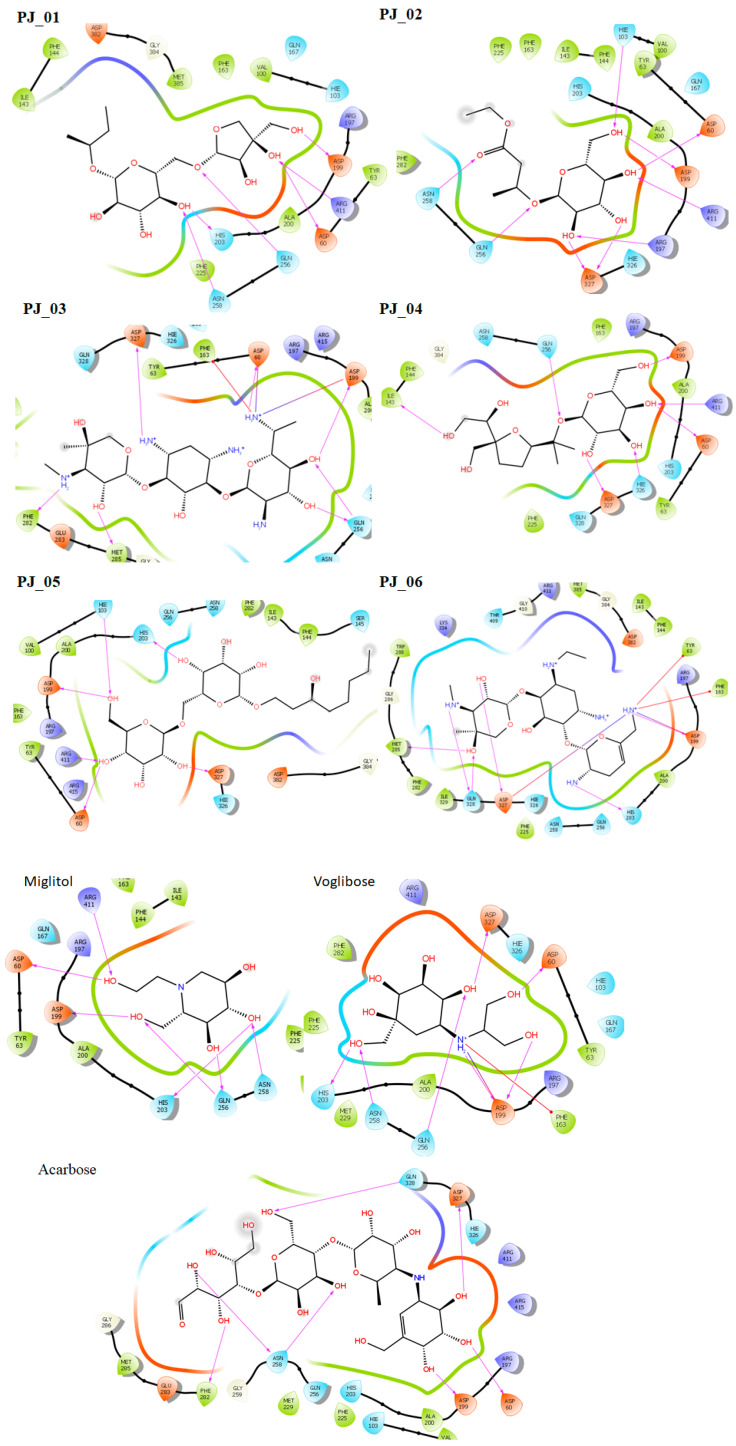
Post-docking 2D interactions of α-glucosidase (PDB ID: 5ZCC)-ligand (PJ_01, PJ_02, PJ_03, PJ_04, PJ_05, PJ_06, and control ligands).

**Figure 2:**
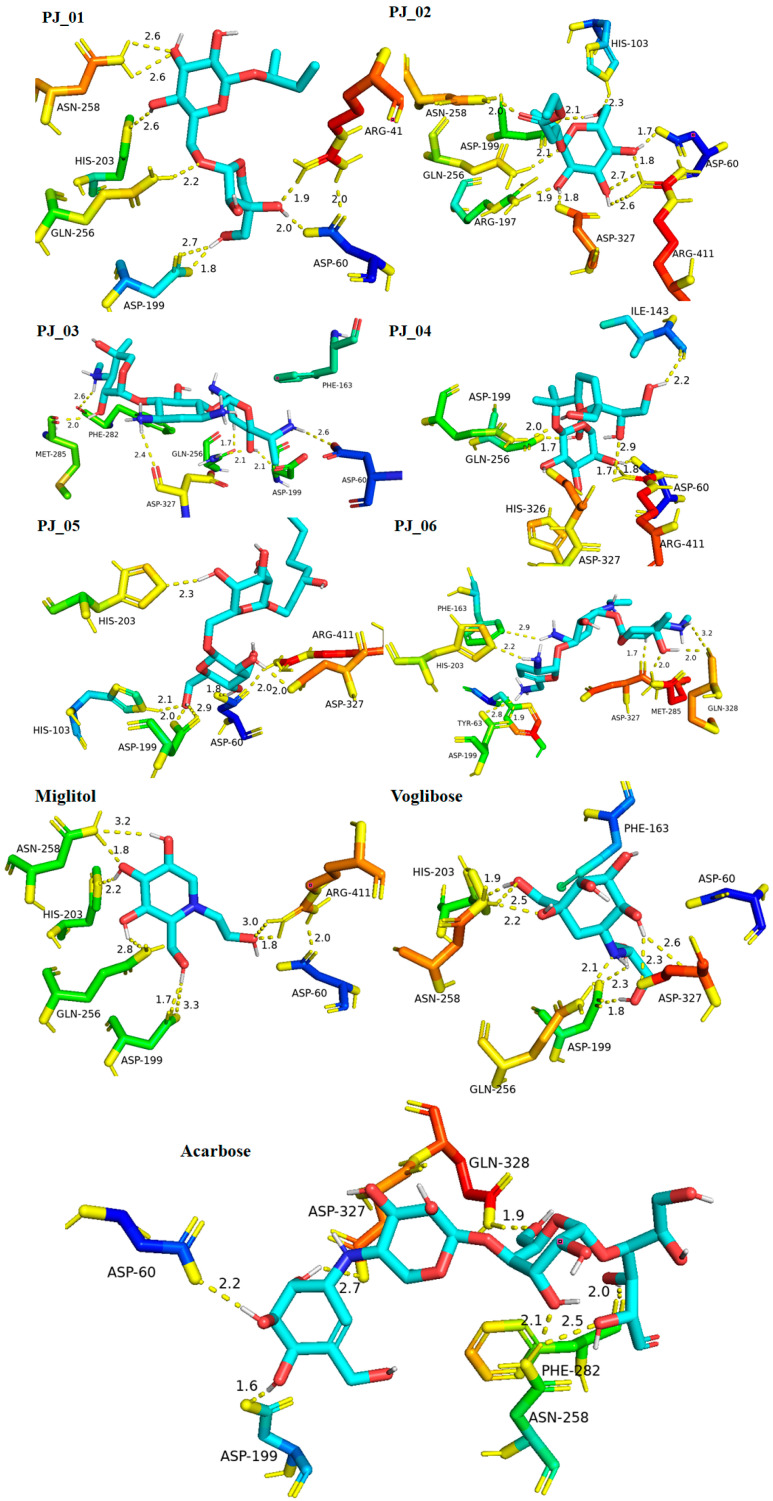
Post-docking protein–ligand 3D interactions of selected compounds (PJ_01, PJ_02, PJ_03, PJ_04, PJ_05, and PJ_06) and control ligands with their interacting distances. The hydrogen-bonding interactions were depicted in a yellow dotted line.

Six compounds were identified from the virtual screening of the seventy-nine compounds of *P. javanica* that exhibited better binding affinity than known α-glucosidase inhibitors. The XP Glide score of these six top hits exceeds those of the approved known α-glucosidase inhibitors, Miglitol and Voglibose. The XP Glide scores of the known α-glucosidase inhibitors Miglitol, Voglibose, and Acarbose were −7.327, −8.662, and −11.975 kcal/mol, respectively. The binding affinities of the top six hits, PJ_01, PJ_02, PJ_03, PJ_04, PJ_05, and PJ_06, were −9.092, −8.579, −13.632, −10.506, −11.013, and −13.818 kcal/mol, respectively. One common method to determine the binding free energy of small organic molecules to receptors is to combine molecular mechanics energies with generalized Born and surface area continuum solvation (MM-GBSA) techniques. The XP glide post-process MM-GBSA dG binding energies predicted by the Prime of known inhibitors Miglitol, Voglibose, and Acarbose were −22.722, −32.823, and −50.035 kcal/mol, respectively. Similarly, glide post-process MM-GBSA dG binding energies of top hits PJ_01, PJ_02, PJ_03, PJ_04, PJ_05, and PJ_06 were −26.259, −36.846, −57.542, −27.704, −22.205, and −80.784 kcal/mol, respectively. The 2D ligand interaction diagram showed that the compound PJ_01 interacts with active site amino acid residues through H-bonding with ASP-60, ASP-199, HIS-203, GLN-256, ASN-258, and ARG-411. The compounds PJ_02 that interact with active site amino acid residues through H-bonding are ASP-60, HIE-103, ARG-197, ASP-199, GLN-256, ASN-258, ASP-327 (2), and ARG-411. The active site-interacting amino acid residues with compound PJ_03 were ASP-60, ASP-199, GLN-256 (2), PHE-282, MET-285, ASP-327 through hydrogen bonding; PHE-163 through π-cation; and ASP-60, ASP-199 through salt bridge. The compound PJ_04 interacts through H-bonding with ASP-60, ILE-143, ASP-196, GLN-256, ASP-326, ASP-327, and ARG-411. The amino acid residues interacting with PJ_05 through H-bonding were ASP-60, HIE-103, ASP-199, HIS-203, ASP-327, and ARG-411. The compound with the highest binding affinity was PJ_06, and the interacting residues were ASP-199, HIS-203, MET-285, ASP-327, and GLN-328, which interact through H-bonding; TYR-63 and PHE-163 interact through π-cation; and ASP-327 interacts via a salt bridge. The 2D interactions of six selected top hits and known inhibitors with α-glucosidase are depicted in Figure 1. Table 3 represents an overview of the types of interactions, the residues associated with each interaction, XP Glide scores, and MM-GBSA dG binding free energies of the top six hits and known inhibitors. Most selected top six hits interact with active site amino acid residues from close distances ≤ 3.0 Å. A visual 3D interaction of selected top hits PJ_01, PJ_02, PJ_03, PJ_04, PJ_05, and PJ_06 and known inhibitors can be seen in Figure 2. All the selected hits are highly soluble in water and violate Lipinski’s rule of five, the Ghose rule, and the Veber rule. Except for compound PJ_02, it does not violate Lipinski’s rule of five. The other important parameter, gastrointestinal absorption of all compounds, was low. The physiochemical parameters of all the molecules are listed in Supplementary Table S1.

### 2.3. Biological Evaluation

#### 2.3.1. In Vitro α-Glucosidase Inhibitory Assay

**Figure 3:**
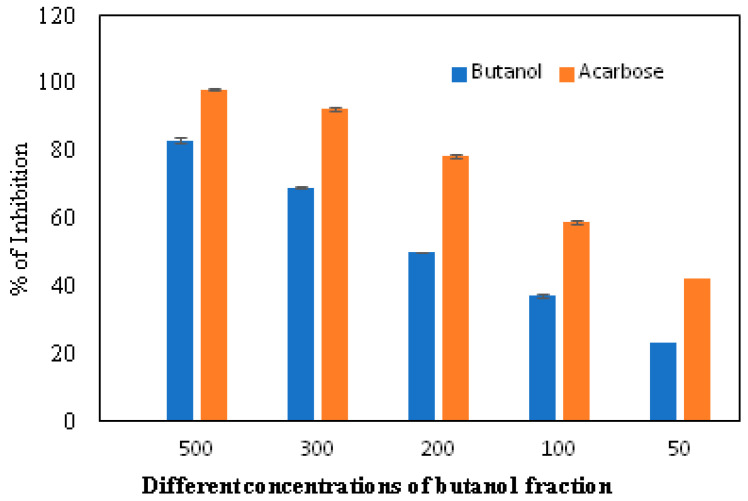
In vitro inhibition of the n-butanol fraction in comparison to the standard inhibitor, Acarbose.

**Figure 4:**
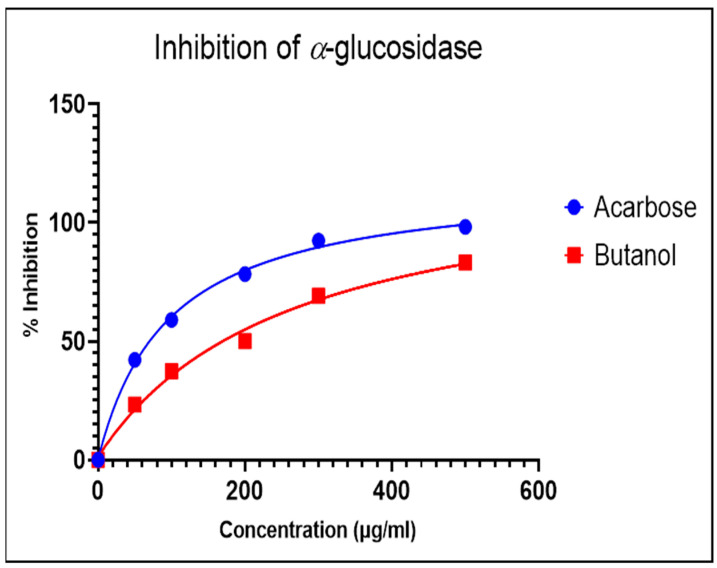
Dose-dependent response curve of the n-butanol fraction and Acarbose.

The α-glucosidase enzyme is one of the essential enzymes of our digestive system, located in the small intestine. It plays an important role in the processing and degradation of complex carbohydrates into small, simple, and absorbable ones. Its inhibition is an effective way to delay absorption and also prevent high postprandial blood glucose levels, which may suppress diabetes progression. The n-butanol fraction showed promising activity in comparison to the known α-glucosidase inhibitor, Acarbose. The α-glucosidase inhibitory activity of the n-butanol fraction of the methanol extract of *P*. *javanica* is shown in Table 4, Figure 3 and Figure 4.

#### 2.3.2. In Vivo Analysis in Swiss Albino Mice

**Figure 5:**
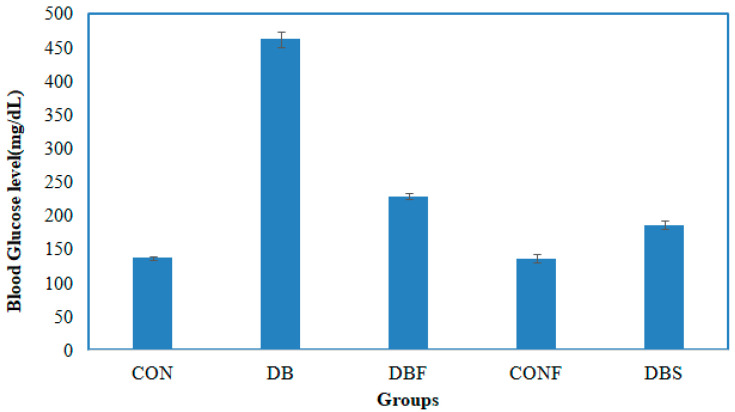
Effect of the n-butanol fraction on the blood glucose level of diabetic mice (CON—control; DB—diabetic mice; DBF—diabetic mice treated with pod extract; CONF—control treated with pod extract; and DBS—diabetes mice treated with standard drug acarbose).

A significant (*p* < 0.01) increased level of blood glucose was noted in the diabetes group of mice in comparison with the control group of mice. In contrast, treatment with the n-butanol fraction of the methanol extract of pods in diabetic mice significantly (*p* < 0.01) decreased the blood glucose levels compared to the diabetes group of mice. The blood glucose levels of control mice (CON), diabetic mice (DB), diabetic mice treated with pod extract (DBF), control mice treated with pod extract (CONF), and diabetes mice treated with the standard drug acarbose (DBS) were 137.33, 462.66, 228.66, 136.66, and 186.33 µg/mL, respectively. The changes in blood glucose levels are shown in Figure 5.

#### 2.3.3. Oxidative Stress Analysis

##### Catalase Activity Assay

**Figure 6:**
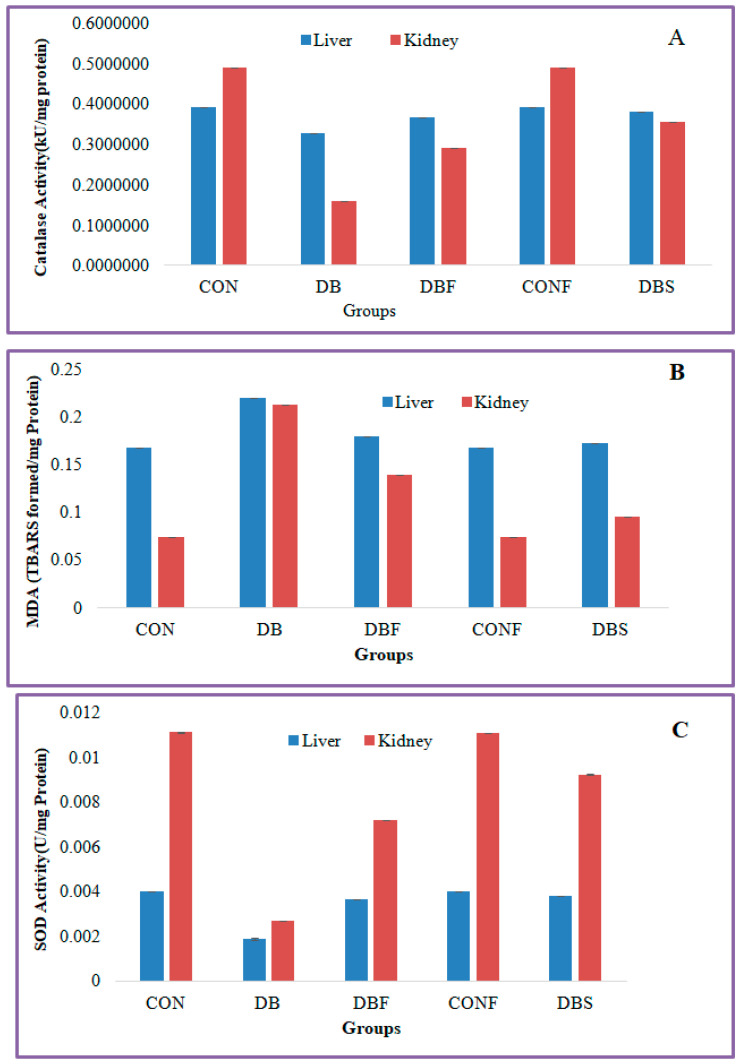
Effect of n-butanol fraction on oxidative stress in the liver and kidney of diabetic mice ((**A**): catalase activity, (**B**): superoxide dismutase, and (**C**): malondialdehyde). CON—control; CONF—control treated with plant fraction; DB—diabetic mice; DBS—diabetic mice treated with standard drug; and DBF—fraction-treated diabetic mice).

The diabetes group of mice showed significantly (*p* < 0.01) decreased levels of catalase activity in the STZ-induced diabetic mice liver and kidney tissues in comparison with the control group of mice, whereas significantly (*p* < 0.01) increased levels of catalase activity were seen in the liver and kidney tissues in extract-treated diabetic mice in comparison to the diabetes group of mice (Figure 6A).

##### MDA Assay

MDA levels were significantly (*p* < 0.01) increased in the liver and kidney tissues of the diabetes group of mice in comparison with the control group of mice, whereas extract-treated diabetic mice showed significantly (*p* < 0.01) decreased levels of MDA in liver and kidney tissues in comparison with the diabetes group of mice (Figure 6B).

##### SOD Activity Assay

A significant (*p* < 0.01) decreased level of SOD activity was found in the liver and kidney tissues of the STZ-induced diabetes group of mice compared to the control group of mice. Treatment with a fraction of the extract in diabetic mice significantly (*p* < 0.01) increased the SOD activity in liver and kidney tissues in comparison with the diabetes group of mice (Figure 6C).

## 3. Discussion

UHPLC-QTOF-MS/MS, renowned for its exceptional selectivity, sensitivity, and accuracy, has been validated as a potent and efficient instrument for conducting metabolomics analysis [43,44,45,46]. Table 1 and Table 2 display detailed information about the retention time, highly accurate precursor ions, molecular formula, and characteristic *m*/*z* of each in both positive and negative ion modes for n-butanol fractions of *P. javanica* methanol extract. A total of 79 chemical components were tentatively identified in the sample compared with authentic samples, data from the available literature, and the obtained MS data. Glycosides, organic carboxylic and sulphonic acids, amides, phenolics, and their derivatives, ester, coumarins, flavonoids, alkaloids, quinine, and a few other types of chemicals were classified based on their structures. The compounds PJ_02, PJ_03, PJ_06, PJ_15, PJ_21, PJ_22, PJ_25, PJ_29, PJ_36, PJ_37, PJ_39, and PJ_55 showed characteristic [M+Na]^+^ ion peaks in the positive ionization mode. The compounds PJ_04, PJ_11, PJ_17, PJ_19, PJ_20, PJ_24, PJ_26, PJ_27, PJ_42, PJ_47, and PJ_51 displayed distinctive [M+NH_4_]^+^ ion peaks in the positive ionization mode. All other compounds showed distinctive [M+H]^+^ ion peaks ranging from PJ_01 to PJ_56. The compounds PJ_58 and PJ_77 were identified in negative ionization mode and showed [M+CH_3_COO]^−^ ion peaks. Under negative ionization mode, distinctive ion peaks corresponding to [M−H]^−^ were observed for compounds from PJ_57 to PJ_79. The compound PJ_61 exhibited an ion peak of [2M+Cl]^−^.

**Figure 7:**
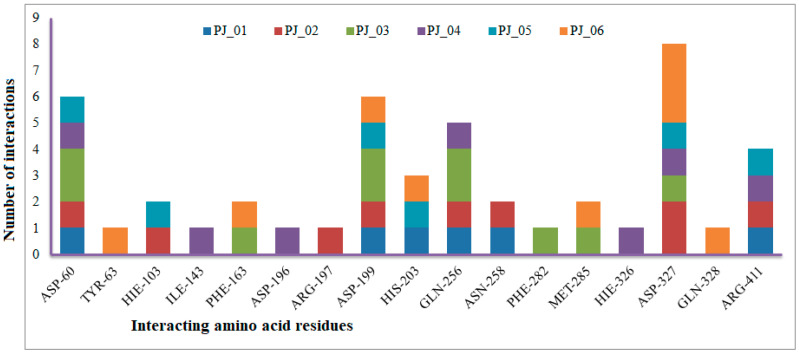
Number of interactions exhibited by selected top six inhibitors (PJ_01, PJ_02, PJ_03, PJ_04, and PJ_06) with active site amino acid residues of α-glucosidase.

**Figure 8:**
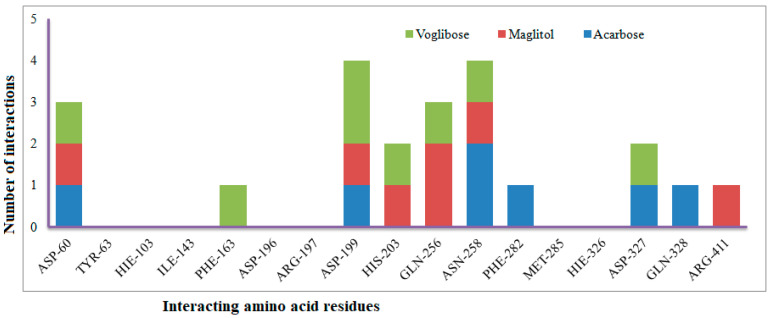
Number of interactions exhibited by selected known α-glucosidase inhibitors Voglibose, Miglitol, and Acarbose with active site amino acid residues.

**Figure 9:**
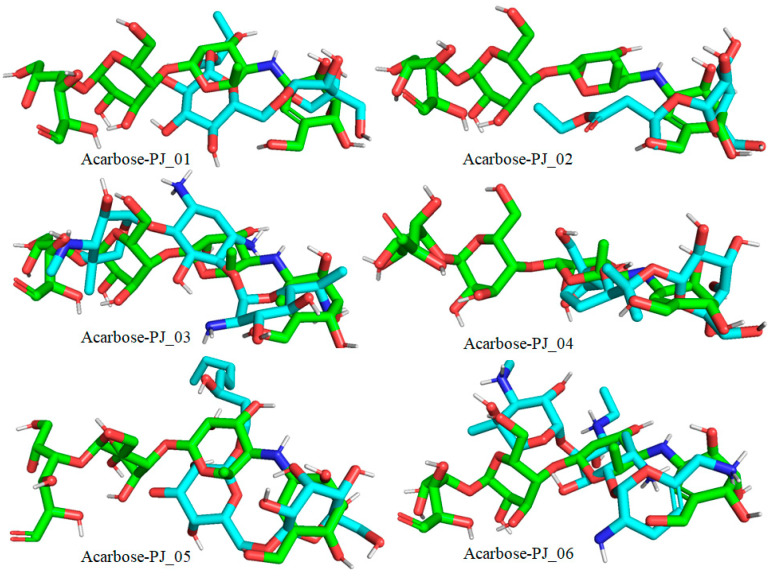
Docked pose of compounds PJ_01, PJ_02, PJ_03, PJ_04, PJ_05, and PJ_06 (cyan color) superimposed on the docked pose of known inhibitor Acarbose (green color) in the active site of α-glucosidase.

**Figure 10:**
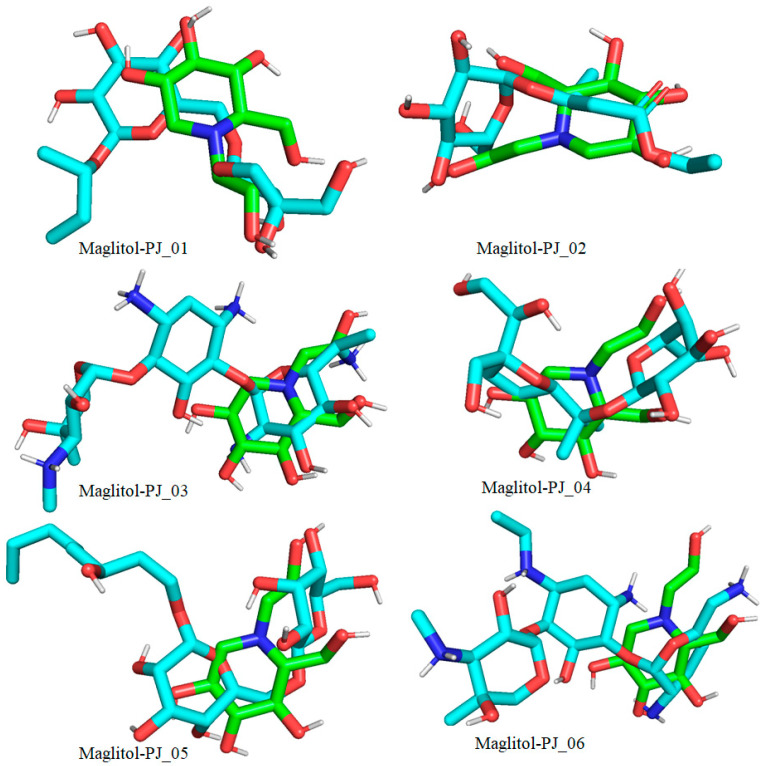
Docked pose of compounds PJ_01, PJ_02, PJ_03, PJ_04, PJ_05, and PJ_06 (cyan color) superimposed on the docked pose of known inhibitor Miglitol (green color) in the active site of α-glucosidase.

**Figure 11:**
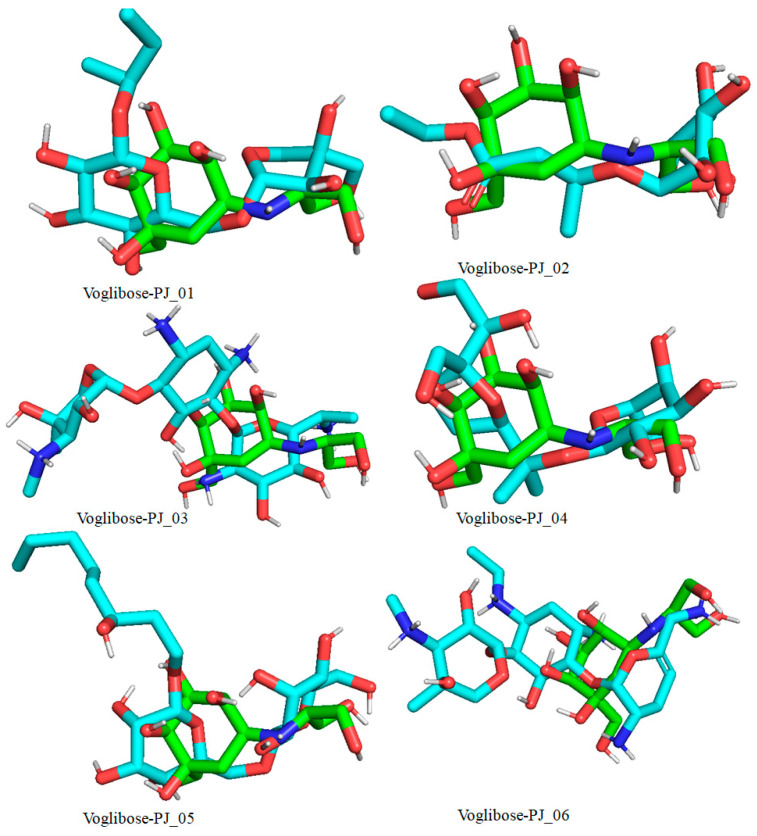
Docked pose of compounds PJ_01, PJ_02, PJ_03, PJ_04, PJ_05, and PJ_06 (cyan color) superimposed on the docked pose of known inhibitor Voglibose (green color) in the active site of α-glucosidase.

The XP Glide scores of Miglitol and Voglibose were −7.327 and −8.662, respectively. The XP Glide scores of hits PJ_01, PJ_02, PJ_03, PJ_04, PJ_05, and PJ_06 were −9.092, −8.579, −13.632, −10.506, −11.013 and −13.818, respectively. The negative sign indicates the energy released after inhibitors bind to α-glucosidase. The XP Glide score of PJ_03 and PJ_05 was also greater than the XP Glide score of the best-known binding inhibitor, Acarbose. All three known inhibitors interact with ASP-60. Similarly, PJ_01, PJ_02, PJ_03, PJ_04, and PJ_05 interact with ASP-60. All known inhibitors exhibit interactions with residues ASP-199 and ASN-258, and PJ_01, PJ_02, PJ_03, PJ_05, and PJ_06 interact with ASP-199, as do PJ_01 and PJ_02 interact with ASN-258. Acarbose and Voglibose showed interactions with ASP-327, as did all other inhibitors except PJ_01. Therefore, most of the selected inhibitors showed similar interactions with known inhibitors. The interactions between known inhibitors and the selected five compounds are shown in Figure 7 and Figure 8. The 3D binding poses of selected hits superimposed on the binding poses of known inhibitors in the active site are shown in Figure 9, Figure 10 and Figure 11. The binding poses of PJ_01, PJ_02, PJ_03, PJ_04, PJ_05, and PJ_06 are similar to all known inhibitors, although it depends on the size of the known inhibitors as well as the size of selected hits. The binding poses of PJ_03 were very close to Acarbose. Similarly, the binding poses of PJ_01, PJ_02, and PJ_04 were very similar to the binding poses of Miglitol and Voglibose.

The compounds PJ_01, PJ_02, PJ_04, and PJ_05 were glycosides in nature, and compounds PJ_03 and PJ_06 were aminoglycosides. The hydrophilic properties of glycoside molecules make them more water-soluble in general [47,48]. All the compounds are highly polar and, therefore, soluble in water. α-Glucosidase inhibition is an effective measure for the management of diabetes mellitus type 2. α-Glucosidase inhibitors reduce the absorbable form of carbohydrates in the small intestine, thus proving effective control against postprandial hyperglycemia. In the present study, the n-butanol fraction of the methanol extract of *P. javanica* pod showed good in vitro α-glucosidase inhibitory activity compared to pure compound Acarbose, a commonly used drug for the medication of type-2 diabetes. The % of α-glucosidase inhibition for n-butanol fraction and acarbose (500 µg/mL) were 83.21 and 98.31, respectively (Table 4). The IC_50_ value of the n-butanol fraction of the extract against α-glucosidase was 261.9 µg/mL, which was 2.7-fold less effective than pure Acarbose. The extract contains a mixture of approx. 79 compounds, of which only 6 exhibited potential in silico binding affinity towards α-glucosidase. Earlier findings also showed that the extract from *Hyoscyamus albus* L. exhibited an inhibitory effect on α-glucosidase, with an IC_50_ of 270.43 µg/mL [49], as the extracts contain a number of compounds. Apart from this, *P. javanica* can be easily found and used as a good food supplement, which could help to prevent T2D management among rural populations. The findings of the present study showed that the blood glucose level of DB mice was 462.66 µg/mL, but the blood glucose level of pod extract-treated mice was 228.66 µg/mL. The blood glucose level of acarbose-treated mice was 186.33 µg/mL, which indicates that the blood glucose level of pod extract-treated mice is comparable with the standard drug acarbose.

The n-butanol fraction not only reduces the blood glucose level in STZ-induced diabetic mice but also reduces oxidative stress in the liver and kidney. STZ-induced type 2 diabetes mellitus is associated with a decrease in catalase expression and a significant reduction in superoxide dismutase activity. However, it elevates polyunsaturated fatty acid oxidation as a result of a substantial increase in MDA in the liver and kidney. The present in vivo investigation revealed that the n-butanol fraction of *P. javanica* pod significantly increased catalase and SOD activities in the liver and kidney tissues in extract-treated diabetic mice; however, MDA levels in the kidney and liver tissue reduced significantly in the extracted-treated diabetic mice group. These findings provide insight into the fact that ingesting n-butanol fractions is relatively safe for developing anti-diabetes formulations from *P*. *javanica* compounds or fractions of extract. Treatment of diabetic mice with 80 mg/Kg/day for 15 consecutive days showed no behavioral changes among the mice.

In vivo oxidative stress analysis showed a significant decrease in MDA levels and an increase in antioxidant enzyme (catalase and SOD) activities in liver and kidney tissue in *Parkia javanica* pod extract-treated diabetic Swiss albino mice. A recent study showed that oral administration of *Parkia speciosa* extract significantly increased the levels of SOD and catalase enzymes and decreased MDA levels in the hepatic and renal tissues of diabetic mice [50]. Recent research also showed that hydrogen peroxide acts as a messenger in the signaling mechanism of insulin secretion, although its higher concentration is toxic. Blood catalase is the principal regulator of H_2_O_2_ metabolism. Generally, blood catalase activity in type 2 diabetes is highly reduced [51]. The oral administration of SOD decreases the blood glucose level in diabetic rats [52]. Poor metabolic control increases the level of MDA, and it acts as a biomarker in type 2 diabetic patients. Our findings showed excellent results by increasing catalase and SOD activity in the liver and kidney, which may be the cause of the glucose level reduction in experimental mice.

It was reported that consumption of one *Parkia roxburghii* pod almost every day has no adverse effect [53]. Although many anti-diabetic drugs are available on the market, the use of natural compounds as hypoglycemic agents is increasing, considering that plant-derived drugs are less toxic. Our results corroborate the previous studies on *P. roxburghii*, *P. speciosa*, and *P. biglobosa* [54,55]. Epigallocatechin gallate and hyperin isolated from *P. roxburghii* and *P. speciosa* pods can potentially inhibit α-glucosidase activity. Aqueous and methanolic extracts of *P. biglobosa* fermented seeds showed hypoglycemic activity in alloxan-induced diabetic rats. Our findings strongly support the findings of in silico binding affinity results in which 79 compounds were identified from the n-butanol fraction of the extract using the UPLC–HRMS technique. Six compounds exhibited excellent binding affinity with the α-glucosidase enzyme compared to known α-glucosidase inhibitors. Another interesting reported fact is that these pods also have anti-cancer, antioxidant, anti-angiogenic, and antiproliferative potential; therefore, these edible pods may be used to treat diabetes as well as cancer after detailed studies of diabetic patients with cancer. The compounds showed poor oral absorption rates in humans, indicating that their potential use as anti-diabetes agents is favorable. The majority of the potential compounds will be excreted from the body after inhibition without being absorbed.

## 4. Materials and Methods

### 4.1. Collection of Plant Material

**Figure 12:**
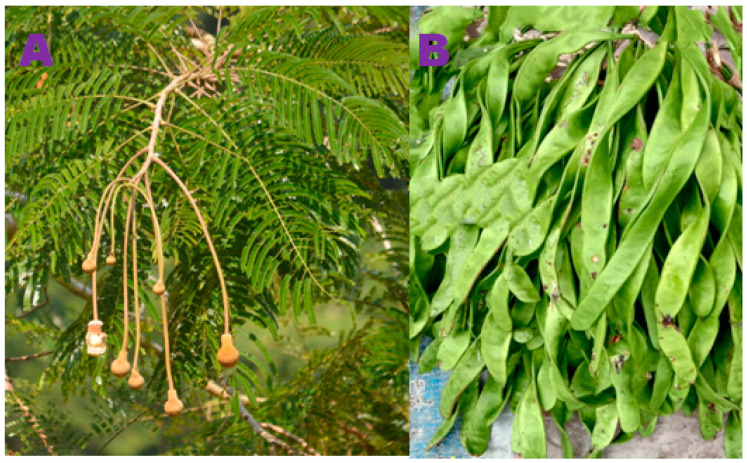
Photographic representation of the tree (**A**) and pods (**B**) of *P. javanica*.

Edible pods of *P. javanica* were procured from the Lake Chowmuhani Market in Agartala, Tripura, India, in March 2023 (Figure 12). The obtained pods have been taken to the Department of Forestry and Biodiversity, Tripura University. The pods were ground into powder, dried without exposure to sunlight, and preserved for further analysis. Primary identification was carried out by Dr. Kausik Majumder, Dept. of Botany, Tripura University. A specimen voucher of the plant (No. BD-01/06) is available in the Central National Herbarium, Government of India, Howrah, Shibpur. The dry powdered pods (0.50 kg) of *P. javanica* were extracted at room temperature using 1 × 5 L MeOH. A sticky mass containing concentrated extract was stored in vacuum desiccators for three days. About 2.0 g of gummy material has been diluted with 50 mL of distilled water and partitioned sequentially with 300 mL of petroleum ether, CHCl_3_, and n-butanol each. The yield of the dried n-butanol fraction was 0.50 g. The known α-glucosidase inhibitors, Acarbose, Miglitol, and Voglibose, are polar in nature. Generally, polar compounds and glycosides like acarbose are available in the n-butanol fraction of plant extracts. The n-butanol-soluble fraction underwent a bioactivity assay and UHPLC-QTOF-MS/MS analysis.

### 4.2. UHPLC-QTOF-MS/MS

The samples were analyzed using the TOF/Q-TOF Mass Spectrometer, Component Name MS Q-TOF, Component Model G6546A, Ion Source Dual AJS ESI, Min Range (*m*/*z*): 100, Max Range (*m*/*z*): 1500, Scan Rate (Spectra/Sec): 1.0. The Q-TOF MS system is conjunct with the UPLC (Agilent Technologies, Santa Clara, CA, USA). A unique collection of tools, such as the G7129B auto sampler, the G7116B column compartment, the G7104A Quat. pumps, the G7117A DAD, and the G6546A MS-Q-TOF, were used to accomplish the LC–MS analysis. A reversed-phase ZORBAX RRHD Eclipse Plus C18 analytical column measuring 100 mm by 2.1 mm by 1.8 µm and running at 40 °C was utilized for the compound separation process. Mobile phase A, which included H_2_O and formic acid (0.1%), and mobile phase B, which included 100% acetonitrile, were used in the elution gradient. The details of solvent composition and timetable with flow rate are given in Table 5 and Table 6, respectively. The solvents (HPLC-grade) methanol, acetonitrile, and formic acid were supplied by Thermo Fisher Scientific (Waltham, MA, USA).

### 4.3. Molecular Docking

All 79 structures of the compound were drawn using ChemDraw Professional 15 and saved in .sdf format. The single .sdf file was imported into Maestro-14 [56], and the molecules were prepared using the LigPrep [57] module, Schrodinger-2022-4. Ligprep retains the original chiralities and ionization states of each input structure while generating a single, low-energy, three-dimensional structure. The energy of the molecules was minimized using the OPLS_2005 force field.

The X-ray crystal structure of PDB IDs of α-glucosidase [58,59] PDB ID: 5ZCC was retrieved from the Research Collaboratory for Structural Bioinformatics (RCSB) data bank, imported into Maestro, and prepared using “Protein Preparation Wizard” [60]. During protein preparation, hydrogens were added to the protein and co-ligand to fill in the missing side chains and missing loops using prime. The energy of the complexes was then minimized using the OPLS_2005 force field. The co-ligand of the active site was selected to generate the receptor grid box of the 15.0 Å cube.

### 4.4. Virtual Screening

The method of structure-based virtual screening was employed to determine the binding affinity of compounds of *P. javanica* to α-glucosidase using Glide [61,62,63,64], Schrodinger-2022-4, LLC, New York, NY, USA. The already prepared molecules are imported into the Virtual Screening Workflow, and the prepared receptor grid is selected. In the VS workflow, 40% of the total molecules from Glide HTVS are forwarded to Glide SP. Then, 40% of the molecules from SP were forwarded to Glide XP, followed by post-processing with prime MM-GBSA. All the computational works were carried out using Dell Inc., System Model: Precision 5820 Tower, Processor: Intel(R) Xeon(R) W-2245 CPU @ 3.90GHz (16 CPUs), ~3.9 GHz, OS: Ubuntu 22.04.1 LTS, 64-bit.

### 4.5. In Vitro α-Glucosidase Enzyme Inhibitory Assays

The assay analysis is performed by following the procedure of Pistia–Brueggeman and Hollingsworth [65]. Moreover, 20 μL of α-glucosidase (5U/mL) were mixed with 100 μL of n-butanol extract at varying concentrations in 250 μL of 0.1 M phosphate buffer (pH 6.8) and incubated at 37 °C for 15 min. Then, 40 μL of 1.0 M pPNG(4-Nitrophenyl-α-D-glucopyranoside) substrate was added and incubated for 45 min at 37 °C for the reaction. For the termination of the reaction, 100 μL of 0.10 N Na_2_CO_3_ was added. The OD value was measured at 405 nm using a spectrophotometer. Acarbose was used as the positive control. The inhibitory activity was calculated using the following formula:

% inhibition of α-glucosidase = (Abs_control_ − Abs_Sample_)/(Abs_control_) × 100

### 4.6. In Vivo Anti-Diabetic Activity

All the experiments with animals and their maintenance were conducted in accordance with institutional practice, within the framework of CCSEA (Committee for Control and Supervision of Experiments on Animals), and as per the Act of the Government of India (2007) for animal welfare. The present study was conducted on Swiss albino mice. The healthy mice of 25.0 g were selected from a mouse colony maintained in the standard laboratory conditions of light (12 h light and 12 h dark), temperature (25.0 ± 2 °C), and humidity (55.0 ± 5%). The experimental mice were kept five per group in polycarbonated cages (43 cm × 27 cm × 14 cm) and fed with mice feed and water ad libitum [66,67].

Mice were intraperitoneally injected with multiple low doses of streptozotocin (STZ) at 50.0 mg/kg body weight for five consecutive days. Freshly prepared 0.10 M citrate buffer (pH 4.5) was used to dissolve the STZ. Experimental mice were kept for 20 h of fasting before STZ administration, and 10% sucrose water was provided during the STZ treatment. The blood was collected from the tail veins of mice to determine the blood glucose level using an Accu-Chek^®^ active (Roche) blood glucose monitoring system at two-day intervals. Experimental mice with blood glucose levels greater than 250 mg/dL were considered to have diabetes [66].

Female mice were divided into control (CON), diabetes (DB), and diabetic mice treated with pod extract (DBF), diabetes mice treated with standard drug acarbose (DBS), and control mice treated with pod extract (CONF) groups (with five mice in each group). The pod extract and acarbose were diluted 100 times in distilled water to prepare the working solution (10 mg of pod extract was dissolved in 1.0 mL of distilled water). Experimental diabetic mice were orally given pod extract (200 µL/mice/day) for 15 consecutive days.

Experimental mice were sacrificed on the 16th day, and blood was collected for blood glucose analysis. The liver and kidney were dissected and kept at −20 °C for oxidative stress analysis.

### 4.7. Blood Glucose Level Analysis

Blood glucose was determined with the help of the ACCU-CHEK^®^ active (Roche) blood glucose monitoring system [66].

### 4.8. Oxidative Stress Analysis

Catalase activity assay: Catalase (CAT; EC 1.11.1.6) activity was determined by following the method [68,69].

SOD activity assay: Superoxide dismutase (SOD; EC 1.15.1.1) activity was assayed following the method [70].

MDA assay: Lipid peroxidation was measured based on the reaction between malondialdehyde and thiobarbituric acid (TBA) following the method [71].

### 4.9. Prediction of Physiochemical Parameters Using SwissADME

SwissADME is a free online tool to assess the pharmacokinetics, drug-likeness, and medicinal chemistry compatibility of small organic molecules [72]. The important physiochemical parameters that were predicted are molecular weight, the numbers of H-bond acceptors and H-bond donors, rotatable bonds, molar refractivity, topological polar surface area, solubility class, gastrointestinal absorption, blood–brain barrier penetration, violation of Lipnskis rule of five, violation of Ghose rule, violation of Veber rule, and bioavailability score [73,74,75,76,77,78].

### 4.10. Statistical Analysis

The statistical analysis of the data was performed with a one-way analysis of variance (ANOVA). The differences were considered significant at *p* < 0.01. Statistical Package for the Social Sciences (SPSS-17) and Microsoft Excel programs were used for calculation and graph preparation.

## 5. Conclusions

The n-butanol fraction of the methanol extract of *P. javanica* pods comprises seventy-nine compounds identified by the UHPLC-QTOF-MS/MS-based chemical profiling technique. Molecular docking investigations of the identified compounds revealed that PJ_01–PJ_06 showed significant binding affinity toward the target protein of diabetes, α-glucosidase. This study also provided evidence that the n-butanol fraction of the pods of *P. javanica* extract has potential in vitro and in vivo anti-diabetic activity. The mice treated with acarbose, mice treated with pod extract, and diabetic mice had blood glucose levels of 186.33 µg/mL, 228.66 µg/mL, and 462.66 µg/mL, respectively. The outcomes of acarbose-treated and pod extract-treated mice are comparable. Our findings suggested that the phytochemicals found in the n-butanol fraction of *P. javanica* have the ability to alleviate hyperglycemia. *P. javanica* is easily available and can serve as a good food supplement, which could help to prevent T2DM among people. In future studies, bio-assay-guided isolation will be conducted to obtain pure active compounds.

## Figures and Tables

**Table 1:** UHPLC-QTOF-MS/MS-based positive mode identifications of compounds; RT, retention time; [M+H]^+^, [M+Na]^+^, and [M+NH_4_]^+^ ions; experimental exact mass; and score.

Comp.	Name of Compound	Formula	RT	Mass	Molecular Ion	Exp. (*m*/*z*)	Score
PJ_01	(2S)-2-butanol-*O*-[β-d-apiofuranosyl-(1→6)-β-d-glucopyranoside]	C_15_H_28_O_10_	6.405	368.1672	[M+H]^+^	369.1750	95.14
PJ_02	Ethyl (S)-3-Hydroxylbutyrate glucoside	C_12_H_22_O_8_	6.584	294.1308	[M+Na]^+^	317.1200	97.44
PJ_03	Antibiotic JI-20B	C_20_H_41_N_5_O_9_	7.532	495.2907	[M+Na]^+^	518.2798	95.52
PJ_04	1,2,10-Trihydroxydihydro-translinalyl oxide 7-*O*-β-d-glucopyranoside	C_16_H_30_O_10_	7.657	382.1832	[M+NH_4_]^+^	400.2170	97.13
PJ_05	(R)-1-*O*-[β-d-glucopyranosyl-(1→6)-β-d-glucopyranoside]-1,3-octanediol	C_20_H_38_O_12_	8.369	470.2353	[M+H]^+^	471.2427	96.93
PJ_06	Netilmicin	C_21_H_41_N_5_O_7_	11.513	475.3007	[M+Na]^+^	498.2896	96.39
PJ_07	C16 Sphinganine	C_16_H_35_NO_2_	12.309	273.2661	[M+H]^+^	274.2734	97.46
PJ_08	Pentanamide	C_5_H_11_NO	12.762	101.0837	[M+H]^+^	102.0910	95.12
PJ_09	Eudesmin	C_22_H_26_O_6_	14.079	386.1721	[M+H]^+^	387.1795	97.68
PJ_10	Phytosphingosine	C_18_H_39_NO_3_	14.128	317.2924	[M+H]^+^	318.2997	98.30
PJ_11	Nonadecanoic acid	C_19_H_38_O_2_	14.382	298.2866	[M+NH_4_]^+^	316.3204	96.37
PJ_12	1-Octadecanamine	C_18_H_39_N	14.547	269.3077	[M+H]^+^	270.3148	96.63
PJ_13	Oleamide	C_18_H_35_NO	14.691	281.2712	[M+H]^+^	282.2785	95.36
PJ_14	1,2-Epoxyhexadecane	C_16_H_32_O	15.163	240.2447	[M+NH_4_]^+^	258.2785	97.28
PJ_15	Estra-1,3,5(10)-triene-3,6β,17β-triol triacetate	C_24_H_30_O_6_	15.721	414.2036	[M+Na]^+^	237.1927	98.72
PJ_16	Militarinone A	C_26_H_37_NO_6_	15.722	459.2612	[M+H]^+^	460.2684	97.35
PJ_17	Arachidic acid	C_20_H_40_O_2_	15.755	312.3020	[M+NH_4_]^+^	330.3358	97.18
PJ_18	4-Hydroxycoumarin	C_9_H_6_O_3_	15.994	162.0314	[M+H]^+^	163.0386	97.35
PJ_19	10-Eicosene	C_20_H_40_	16.352	280.3124	[M+NH_4_]^+^	298.3462	96.63
PJ_20	Tetrahydropersin	C_23_H_44_O_4_	16.485	384.3232	[M+NH_4_]^+^	402.3569	97.74
PJ_21	Rutamarin	C_21_H_24_O_5_	16.695	356.1617	[M+Na]^+^	379.1510	97.78
PJ_22	Indicumenone	C_15_H_24_O_3_	16.885	252.1721	[M+Na]^+^	275.1613	98.85
PJ_23	2,6-вimethoxy-4-propylphenol	C_11_H_16_O_3_	16.885	196.109	[M+H]^+^	197.1169	99.39
PJ_24	Orysastrobin	C_18_H_25_N_5_O_5_	17.190	391.1854	[M+NH_4_]^+^	409.2192	96.30
PJ_25	Tetraneurin A	C_17_H_22_O_6_	17.376	322.1409	[M+Na]^+^	345.1301	97.31
PJ_26	2-Pentadecylfuran	C_19_H_34_O	17.398	278.2604	[M+NH_4_]^+^	296.2942	96.63
PJ_27	Docosanoic acid	C_22_H_44_O_2_	17.430	340.3334	[M+NH_4_]^+^	358.3673	97.85
PJ_28	Dimethylbenzyl carbinyl hexanoate	C_16_H_24_O_2_	18.548	248.1773	[M+H]^+^	249.1844	97.96
PJ_29	7-Methoxy-2,2,4-trimethyl-3-(4- methoxyphenyl)-2H-1-benzopyran	C_20_ H_22_O_3_	18.867	310.1561	[M+Na]^+^	333.1454	97.24
PJ_30	3-Butylidene-7-hydroxyphthalide	C_12_H_12_O_3_	20.060	204.0783	[M+H]^+^	205.0856	99.29
PJ_31	Diisobutyl phthalate	C_16_H_22_O_4_	20.060	278.1514	[M+H]^+^	279.1585	99.27
PJ_32	18-Oxooleate	C_18_H_32_O_3_	20.266	296.2344	[M+H]^+^	297.2417	96.54
PJ_33	Decahydro-2-naphthoic acid	C_11_H_18_O_2_	20.267	182.1302	[M+H]^+^	183.1375	98.69
PJ_34	(+)-12-(2-Cyclopenten-1-yl)-2-dodecanone	C_17_H_30_O	20.271	250.2291	[M+H]^+^	251.2363	97.87
PJ_35	5β-gonane	C_17_H_28_	20.639	232.2188	[M+H]^+^	233.2259	97.22
PJ_36	Gingerdione	C_17_H_24_O_4_	21.139	292.1668	[M+Na]^+^	315.1560	95.61
PJ_37	2,3-dihydroxycyclopentane-undecanoic acid	C_16_H_30_O_4_	21.686	286.2137	[M+Na]^+^	309.2029	95.93
PJ_38	1,3-Octadiene	C_8_H_14_	21.689	110.1093	[M+H]^+^	111.1165	95.95
PJ_39	Acetyl tributyl citrate	C_20_H_34_O_8_	21.842	402.2246	[M+Na]^+^	425.2138	97.76
PJ_40	Palmitic amide	C_16_H_33_ NO	22.714	255.2555	[M+H]^+^	256.2628	97.70
PJ_41	Drotaverine	C_24_H_31_NO_4_	22.916	397.2245	[M+H]^+^	398.2318	98.23
PJ_42	Resolvin D2	C_22_H_32_O_5_	23.433	376.2241	[M+NH_4_]^+^	394.2579	97.29
PJ_43	Talatizamine	C_24_H_39_NO_5_	23.435	421.2819	[M+H]^+^	422.2892	97.76
PJ_44	Bombykol	C_16_H_30_O	23.490	238.2290	[M+H]^+^	239.2363	98.05
PJ_45	1-Monopalmitin	C_19_H_38_O_4_	23.492	330.2762	[M+H]^+^	331.2835	96.82
PJ_46	Dubamine	C_16_H_11_NO_2_	23.587	249.078	[M+H]^+^	250.0856	97.08
PJ_47	Tetrahydro-6-(2-hydroxy-16,19- dimethylhexacosyl)-4-methyl-2H-pyran-2-one	C_34_H_66_O_3_	24.854	522.4998	[M+NH_4_]^+^	540.5337	95.73
PJ_48	Stearamide	C_18_H_37_NO	25.150	283.2866	[M+H]^+^	284.2939	95.51
PJ_49	Glycidyl stearate	C_21_H_40_O_3_	25.768	340.2969	[M+H]^+^	341.3042	96.11
PJ_50	2-Tetradecylcyclobutanone	C_18_H_34_O	25.771	266.2604	[M+H]^+^	267.2676	97.67
PJ_51	4,4′-Diapophytoene	C_30_H_48_	28.099	408.3746	[M+NH_4_]^+^	426.4084	96.41
PJ_52	Bis(4-methoxybenzoyl)-3a,29-dihydroxy-8-multifloren-7-one	C_46_H_60_O_7_	29.572	724.4353	[M+H]^+^	725.4425	97.07
PJ_53	Pentalen-13-al	C_15_H_22_O	32.372	218.1665	[M+H]^+^	219.1738	98.19
PJ_54	1-Octadecanamine	C_18_H_39_N	32.465	269.3076	[M+H]^+^	270.3149	97.94
PJ_55	Dexpanthenol	C_9_H_19_NO_4_	32.515	205.1308	[M+Na]^+^	228.1200	97.87
PJ_56	Capsi-amide	C_17_H_35_NO	32.598	269.2712	[M+H]^+^	270.2785	96.54

**Table 2:** UHPLC-QTOF-MS/MS-based negative mode identifications of compounds; RT, retention time; [M+H]^−^, [M+CH_3_COO]^+^, and [2M+Cl]^−^; experimental exact mass; and score.

Comp.	Name of Compound	Formula	RT	Mass	Molecular Ion	*m*/*z*	Score
PJ_57	8-Demethyltetracenomycin C	C_22_H_18_O_11_	5.630	458.0846	[M−H]^−^	457.0772	99.05
PJ_58	Tecostanine	C_11_H_21_NO	11.216	183.1620	[M+CH_3_COO]^−^	242.1759	99.10
PJ_59	2-ethoxy-5-(1-propenyl)-phenol	C_11_H_14_O_2_	12.314	178.0993	[M−H]^−^	177.0920	98.11
PJ_60	2,3-Dihydro-3-hydroxy-6-methoxy-2,2-dimethyl-4H-1-benzopyran-4-one	C_12_H_14_O_4_	12.315	222.0891	[M−H]^−^	221.0818	99.85
PJ_61	Tropolone	C_7_H_6_O_2_	12.315	122.0367	[2M+Cl]^−^	279.0403	99.95
PJ_62	(±)-1,4-nonanediol diacetate	C_13_H_24_O_4_	13.236	244.1672	[M−H]^−^	243.1599	97.65
PJ_63	9,10-dihydroxy stearic acid	C_18_H_36_O_4_	16.578	316.2611	[M−H]^−^	315.2538	98.99
PJ_64	3-hydroxyestra-1,3,5(10)-trien-16-one	C_18_H_22_O_2_	17.965	270.1618	[M−H]^−^	269.1545	98.53
PJ_65	18-hydroxy-9*Z*-octadecenoic acid	C_18_H_34_O_3_	19.040	298.2505	[M−H]^−^	297.2433	98.44
PJ_66	Lauric acid	C_12_H_24_O_2_	19.792	200.1774	[M−H]^−^	199.1701	97.13
PJ_67	Lauryl hydrogen sulfate	C_12_H_26_O_4_S	20.253	266.1548	[M−H]^−^	265.1476	97.22
PJ_68	(R)-10-hydroxystearic acid	C_18_H_36_O_3_	20.265	300.2661	[M−H]^−^	299.2588	98.44
PJ_69	Hexestrol monomethyl ether	C_19_H_24_O_2_	20.362	284.1773	[M−H]^−^	283.1700	98.83
PJ_70	16-Hydroxy hexadecanoic acid	C_16_H_32_O_3_	22.198	272.2348	[M−H]^−^	271.2276	97.29
PJ_71	Myristic acid	C_14_H_28_O_2_	22.458	228.2088	[M−H]^−^	227.2015	98.77
PJ_72	12α-methylpregna-4,9(11)-diene-3,20-dione	C_22_H_30_O_2_	22.529	326.2241	[M−H]^−^	325.2169	97.98
PJ_73 *	9α-(3-Methyl-2E-butenoyloxy)-4S-hydroxy-10(14)-oplopen-3-one 4-acetate	C_22_H_32_O_5_	23.341	376.2246	[M−H]^−^	375.2173	98.56
PJ_74	4,8,12-trimethyltridecanoic acid	C_16_H_32_O_2_	24.773	256.2400	[M−H]^−^	255.2328	99.81
PJ_75	11-cycloheptylundecanoic acid	C_18_H_34_O_2_	25.196	282.2558	[M−H]^−^	281.2484	98.13
PJ_76	Nonyl octanoate	C_17_H_34_O_2_	25.472	270.2556	[M−H]^−^	269.2484	98.27
PJ_77	Tetradecyl sulfate	C_14_ H_30_ O_4_S	26.072	294.1862	[M+CH_3_COO]^−^	353.2002	97.62
PJ_78	Docusate	C_20_H_38_O_7_ S	26.425	422.2335	[M−H]^−^	421.2262	95.97
PJ_79	Stearic acid	C_18_ H_36_O_2_	26.671	284.2715	[M−H]^−^	283.2642	98.24

* https://hmdb.ca/metabolites/HMDB0040956 (accessed on 7 July 2024).

**Table 3:** Extra precision Glide score (XPGS), MM-GBSA dG-binding free energies, and interacting amino acid residues of α-glucosidase with the top six selected hits.

Compound Number	XPGS	MMGBSA dG Bind	Interacting Amino Acid Residues
PJ_01	−9.092	−26.259	H-bonding: ASP-60, ASP-199, HIS-203, GLN-256, ASN-258, and ARG-411
PJ_02	−8.579	−36.846	H-bonding: ASP-60, HIE-103, ARG-197, ASP-199, GLN-256, ASN-258, ASP-327 (2), and ARG-411
PJ_03	−13.632	−57.542	H-bonding: ASP-60, ASP-199, GLN-256 (2), PHE-282, MET-285, and ASP-327 Π-cation: PHE-163 Salt bridge: ASP-60 and ASP-199
PJ_04	−10.506	−27.704	H-bonding: ASP-60, ILE-143, ASP-196, GLN-256, ASP-326, ASP-327, and ARG-411
PJ_05	−11.013	−22.205	H-bonding: ASP-60, HIE-103, ASP-199, HIS-203, ASP-327, and ARG-411
PJ_06	−13.818	−80.784	H-bonding: ASP-199, HIS-203, MET-285, ASP-327, and GLN-328 Π-cation: TYR-63 and PHE-163 Salt bridge: ASP-327
Maglitol	−7.327	−22.722	H-bonding: ASP-60, ASP-199, HIE-203, GLN-256 (2), ASN-258, and ARG-411
Voglibose	−8.662	−32.823	H-bonding: ASP-60, ASP-199, HIS-203, GLN-256, ASN-258, and ASP-327 Π-cation: PHE-163 Salt bridge: ASP-199
Acarbose	−11.975	−54.124	H-bonding: ASP-60, ASP-199, ASN-258, PHE-282, ASP-327, and GLN-328

**Table 4:** Inhibition of α-glucosidase by n-butanol fraction and known inhibitor, Acarbose.

Fraction	% of α-Glucosidase Inhibition					IC_50_ (µg/mL)
	50 (µg/mL)	100 (µg/mL)	200 (µg/mL)	300 (µg/mL)	500 (µg/mL)	
Butanol	23.31±0.51	37.38 ± 0.20	50.11 ± 0.34	69.23 ± 0.73	83.21 ± 0.21	261.9
Acarbose	42.15 ± 0.36	59.02 ± 0.60	78.34 ± 0.37	92.48 ± 0.50	98.31 ± 0.35	95.98

Values are the mean ± SD of three parallel measurements; *p* value < 0.0001; and results are statistically significant.

**Table 5:** Solvent (water and acetonitrile) composition during HPLC.

Sl.No	Channel	Ch.1 Solvent	Name 1	Used	Percent
1	A	100.0% Water V.03	0.1% Formic acid	Yes	95.0
2	B	100.0% Acetonitrile V.03	-	Yes	5.0

**Table 6:** Timetable, solvent composition, and flow rate during HPLC.

Sl.No	Time (min)	A (%)	B (%)	C (%)	D (%)	Flow (mL/min)	Pressure (Bar)
1	Start. cond	95.0	5.0	0.0	0.0	0.30	1300.0
2	25.00	0.0	100.0	0.0	0.0	0.30	1300.0
3	30.00	0.0	100.0	0.0	0.0	0.30	1300.0
4	31.00	95.0	5.0	0.0	0.0	0.30	1300.0
5	35.00	95.0	5.0	0.0	0.0	0.30	1300.0

## Data Availability

The original contributions presented in the study are included in the article/Supplementary Material, further inquiries can be directed to the corresponding authors.

## Supplementary Materials

The following supporting information can be downloaded at: https://www.mdpi.com/article/10.3390/ph17070968/s1. Figure S1: UHPLC-QTOF-MS/MS-based spectra of compounds identified by positive ionization mode; Figure S2: UHPLC-QTOF-MS/MS-based spectra of compounds identified by negative ionization mode; Figure S3: Structure of compounds identified by UPLC–HRMS in both positive and negative ionization mode; Table S1: Physiochemical parameters of PJ_01, PJ_02, PJ_03, PJ_04, PJ_05, and PJ_06 inhibitors predicted by SwissADME.
