# Supplementary material for: Parkia javanica Edible Pods Reveal Potential as an Anti-Diabetic Agent: UHPLC-QTOF-MS/MS-Based Chemical Profiling, In Silico, In Vitro, In Vivo, and Oxidative Stress Studies
Source: Pharmaceuticals (Basel). 2024 Jul 21;17(7):968. doi: 10.3390/ph17070968 (PMC11280426; doi:10.3390/ph17070968)
Supplement: Supplementary file 1 [file pharmaceuticals-17-00968-s001.zip › pharmaceuticals-3084402-supplementary.pdf]

Article

Parkia javanica Edible Pods Reveal Potential as an Anti-Diabetic Agent: UHPLC-QTOF-MS/MS-Based Chemical Profiling, In Silico, In Vitro, In Vivo, and Oxidative Stress Studies

Alekhya Sarkar [1], Arjita Chakrabarti [2], Samhita Bhaumik [3], Bimal Debnath [1], Shiv Shankar Singh [2], Rajat Ghosh [4], Magdi E. A. Zaki [5,*], Sami A. Al-Hussain [5] and Sudhan Debnath [6,*]

[1] Department of Forestry and Biodiversity, Tripura University, Suryamaninagar 799022, India; alekhya.sarkar@gmail.com (A.S.); bimaldebnath@tripurauniv.ac.in (B.D.)
[2] Department of Zoology, Tripura University, Suryamaninagar 799022, India; arjitachakrabarti95@gmail.com (A.C.); shivssingh@tripurauniv.ac.in (S.S.S.)
[3] Department of Chemistry, Women's College, Agartala 799001, India; samhitabhaumik@gmail.com
[4] In Silico Drug Design Lab., Department of Pharmacy, Tripura University, Suryamaninagar 799022, India; rajatghosh@tripurauniv.ac.in
[5] Department of Chemistry, Faculty of Science, Imam Mohammad Ibn Saud Islamic University, Riyadh 11623, Saudi Arabia; sahussain@imamu.edu.sa
[6] Department of Chemistry, Netaji Subhash Mahavidyalaya, Udaipur 799114, India
* Correspondence: mezaki@imamu.edu.sa (M.E.A.Z.); bcsdebnath@gmail.com (S.D.)

Sl.No	Contents	Page No
1.	**Table S1**. Physiochemical parameters of PJ_01, PJ_02, PJ_03, PJ_04, PJ_05 and PJ_06 inhibitors	02
2.	**Figure S1**. Spectra of compounds indentified by positive ionization mode	03-13
3.	**Figure S2**. UPLC–HRMS Spectra of compounds indentified by negative ionization mode	14-18
4.	**Figure S3.** Structure of Compounds identified by UPLC–HRMS both positive and negative mode of ionization.	19-24

Table S1. Physiochemical parameters of PJ_01, PJ_02, PJ_03, PJ_04, PJ_05 and PJ_06 inhibitors predicted by SwissADME

Parameters	PJ_01	PJ_02	PJ_03	PJ_04	PJ_05	PJ_06
MW	368.38	294.30	495.57	382.40	475.30	475.58
NRB	7	7	6	7	-	8
NHA	10	8	14	10	-	12
NHD	6	4	10	7	-	8
MR	81.34	65.79	115.73	86.26	-	117.83
TPSA (A^2)	158.30	125.68	254.18	169.30	-	199.73
iLOGp	1.65	1.72	1.73	1.92	-	2.87
SC in water	Very Soluble	Very soluble	Highly soluble	Highly soluble	-	Highly soluble
GI	Low	Low	Low	Low	-	Low
BBB	No	No	No	No	-	No
vROF	1	0	2	1	-	2
vGhose	1	1	3	1	-	2
vVeber	1	1	1	1	-	1

MW: Molecular weight, NHA: No of H-bond acceptor, NHD: No of H-bond donor, NRB: No. of rotatable bonds, MR: Molar refractivity, TPSA: Topological polar surface area, SC: Solubility class, GI: Gastrointestinal absorption, BBB: Blood Brain Barrier Penetraton, vROF: Violation of Lipnskis rule of five, vGhose: Violation of Ghose rule, vVeber: Violation of veber rule, BS: Bioavailability score.

<table>
<tr><td colspan="2">Figure S1. UPLC–HRMS Spectra of compounds indentified by positive ionization mode</td></tr>
<tr><td>Cpd</td><td>Spectra</td></tr>
<tr><td>1</td><td>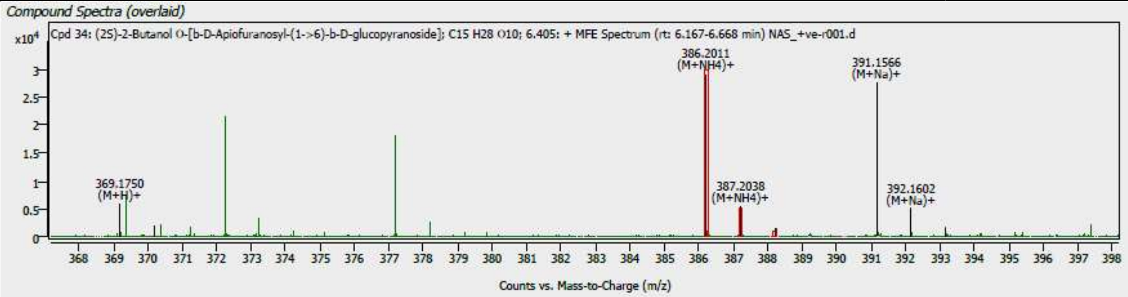
</td></tr>
<tr><td>2</td><td>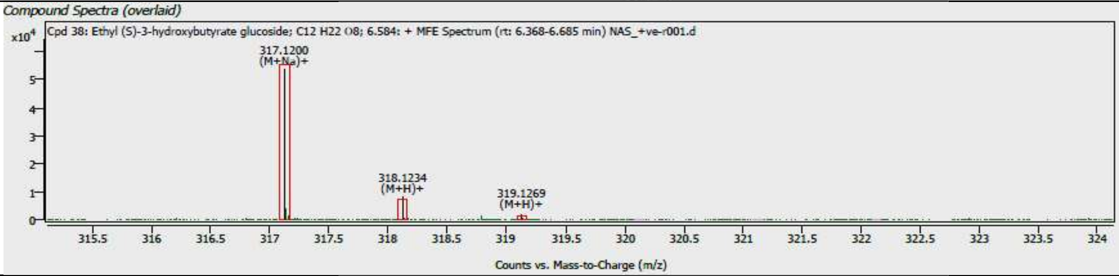
</td></tr>
<tr><td>3</td><td>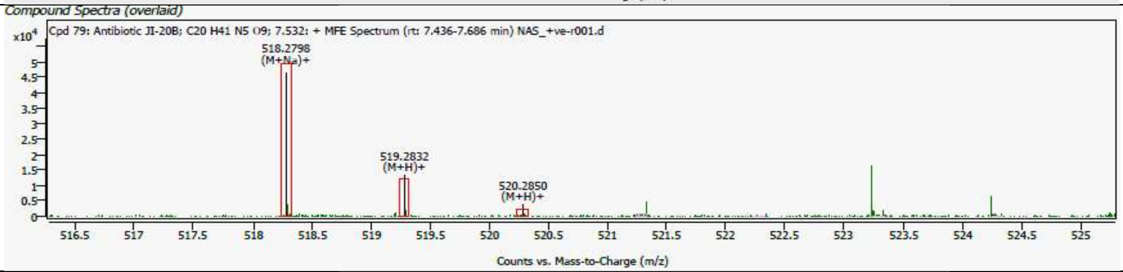
</td></tr>
<tr><td>4</td><td>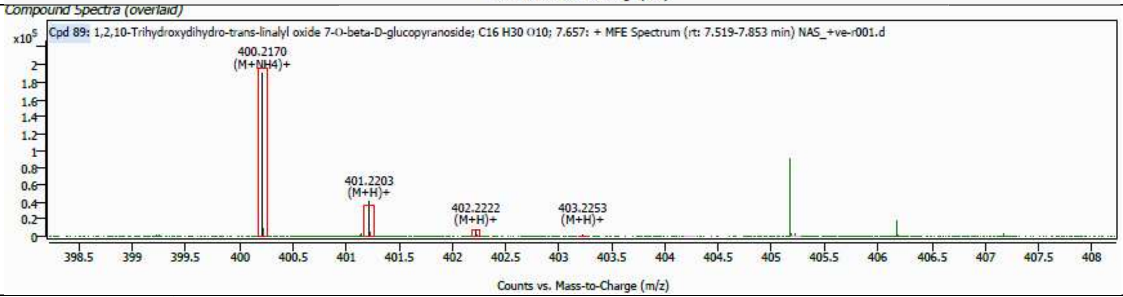
</td></tr>
<tr><td>5</td><td>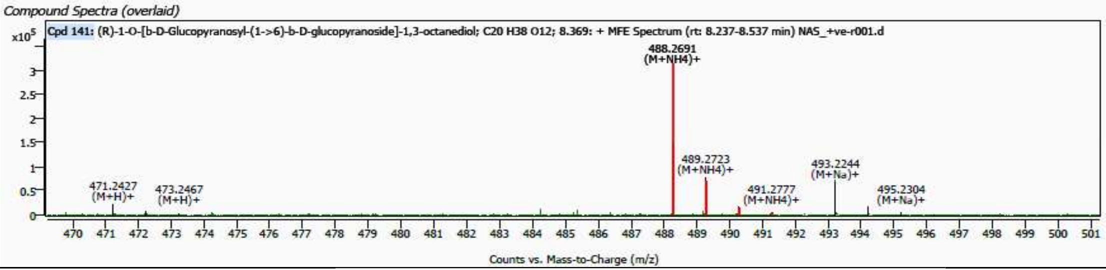
</td></tr>
</table>

6

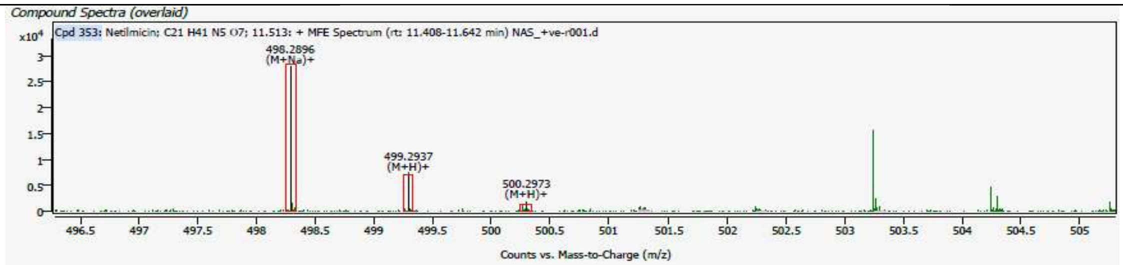
Compound Spectra (overlaid)
Cpd 353: Netilmicin; C21 H41 N5 O7; 11.513: + MFE Spectrum (rt: 11.408-11.642 min) NAS_+ve-r001.d
x10^4
498.2896
(M+Na)+
499.2937
(M+H)+
500.2973
(M+H)+
Counts vs. Mass-to-Charge (m/z)

7

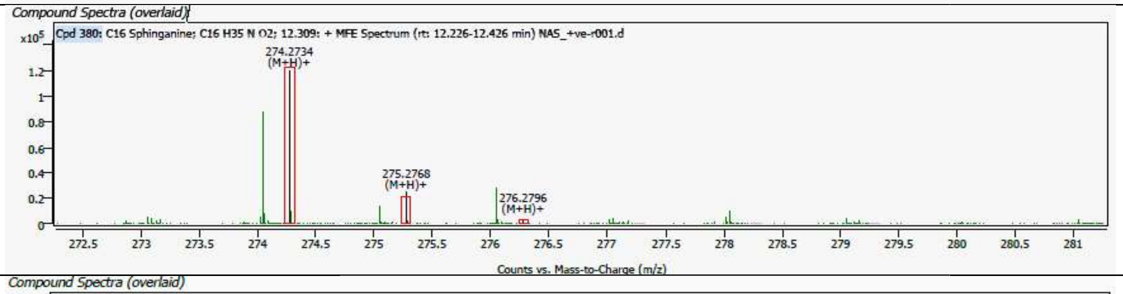
Compound Spectra (overlaid)
Cpd 380: C16 Sphinganine; C16 H35 N O2; 12.309: + MFE Spectrum (rt: 12.226-12.426 min) NAS_+ve-r001.d
x10^5
274.2734
(M+H)+
275.2768
(M+H)+
276.2796
(M+H)+
Counts vs. Mass-to-Charge (m/z)

8

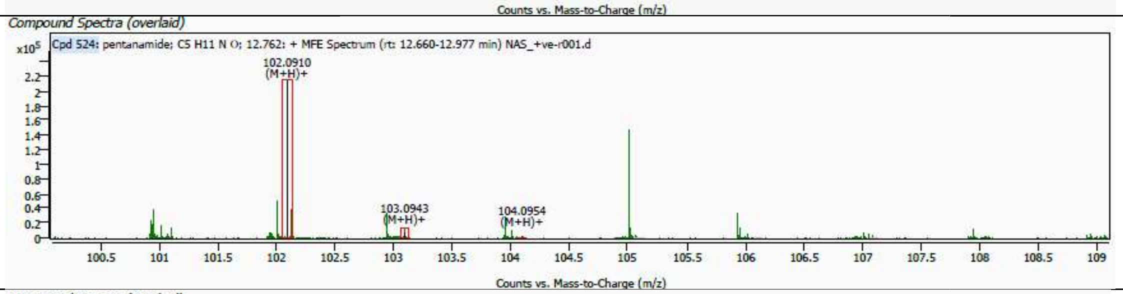
Compound Spectra (overlaid)
Cpd 524: pentanamide; C5 H11 N O; 12.762: + MFE Spectrum (rt: 12.660-12.977 min) NAS_+ve-r001.d
x10^5
102.0910
(M+H)+
103.0943
(M+H)+
104.0954
(M+H)+
Counts vs. Mass-to-Charge (m/z)

9

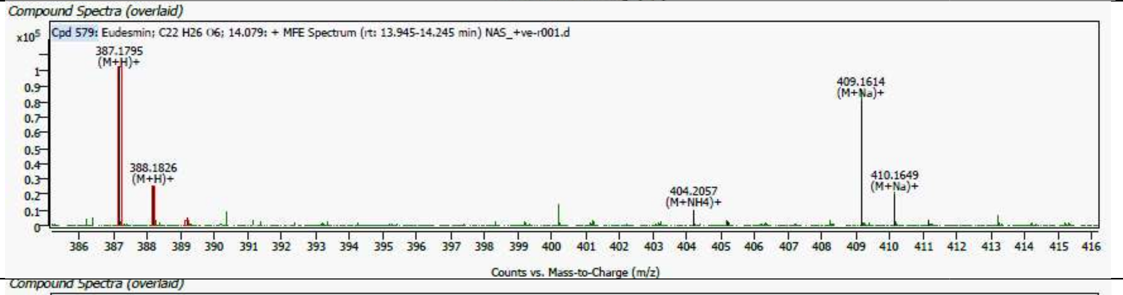
Compound Spectra (overlaid)
Cpd 579: Eudesmin; C22 H26 O6; 14.079: + MFE Spectrum (rt: 13.945-14.245 min) NAS_+ve-r001.d
x10^5
387.1795
(M+H)+
388.1826
(M+H)+
404.2057
(M+NH4)+
409.1614
(M+Na)+
410.1649
(M+Na)+
Counts vs. Mass-to-Charge (m/z)

10

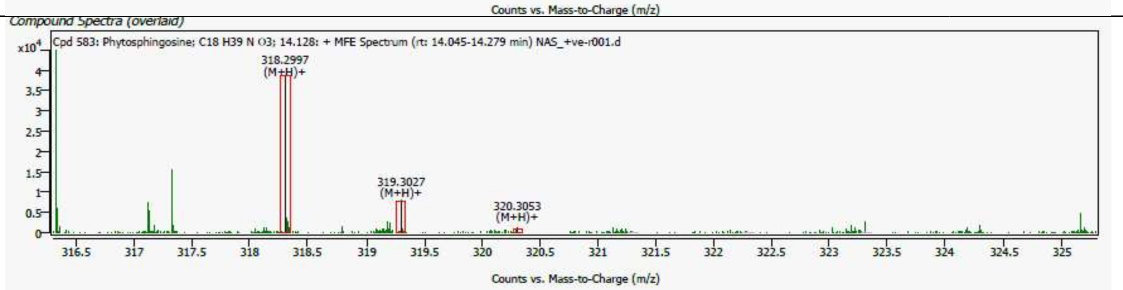
Compound Spectra (overlaid)
Cpd 583: Phytosphingosine; C18 H39 N O3; 14.128: + MFE Spectrum (rt: 14.045-14.279 min) NAS_+ve-r001.d
x10^4
318.2997
(M+H)+
319.3027
(M+H)+
320.3053
(M+H)+
Counts vs. Mass-to-Charge (m/z)

11

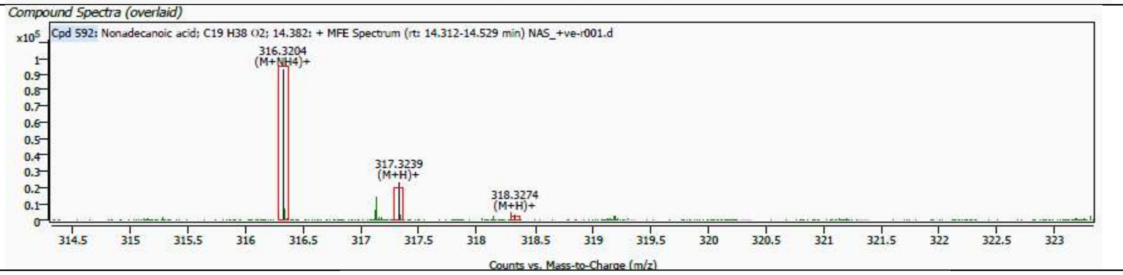
Compound Spectra (overlaid)
Cpd 592: Nonadecanoic acid; C19 H38 O2; 14.382: + MFE Spectrum (rt: 14.312-14.529 min) NAS_+ve-r001.d
x10^5
316.3204
(M+NH4)+
317.3239
(M+H)+
318.3274
(M+H)+
Counts vs. Mass-to-Charge (m/z)

12

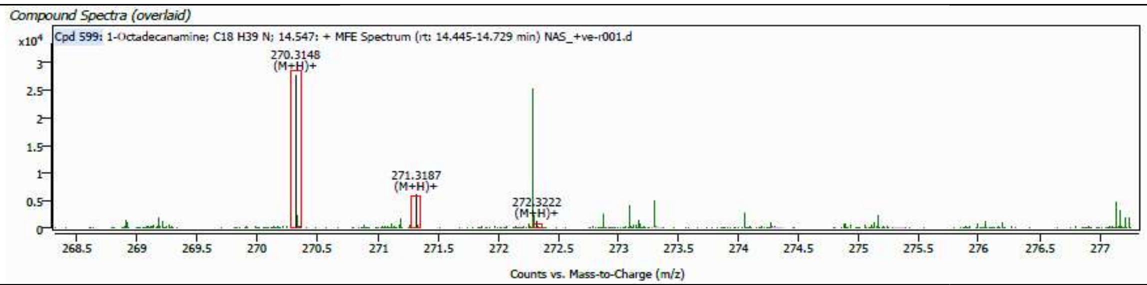
Compound Spectra (overlaid)
Cpd 599: 1-Octadecanamine; C18 H39 N; 14.547: + MFE Spectrum (rt: 14.445-14.729 min) NAS_+ve-r001.d
x10^4
270.3148
(M+H)+
271.3187
(M+H)+
272.3222
(M+H)+
Counts vs. Mass-to-Charge (m/z)

13

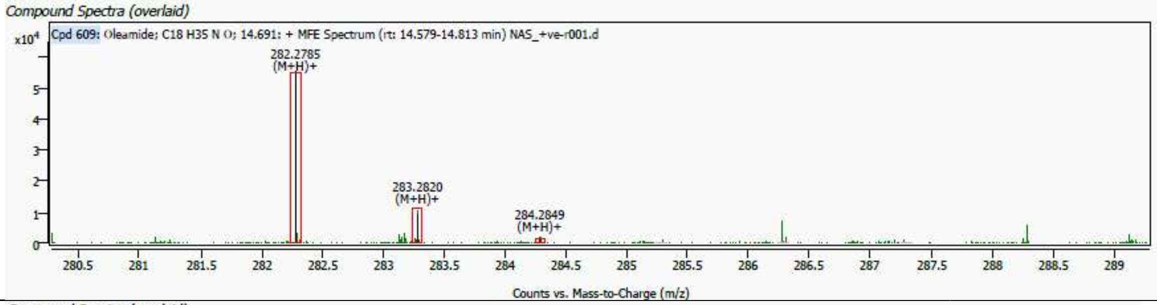
Compound Spectra (overlaid)
Cpd 609: Oleamide; C18 H35 N O; 14.691: + MFE Spectrum (rt: 14.579-14.813 min) NAS_+ve-r001.d
x10^4
282.2785
(M+H)+
283.2820
(M+H)+
284.2849
(M+H)+
Counts vs. Mass-to-Charge (m/z)

14

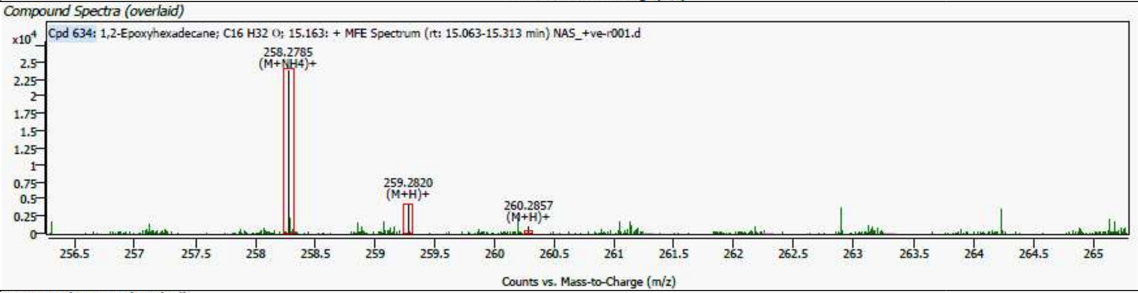
Compound Spectra (overlaid)
Cpd 634: 1,2-Epoxyhexadecane; C16 H32 O; 15.163: + MFE Spectrum (rt: 15.063-15.313 min) NAS_+ve-r001.d
x10^4
258.2785
(M+NH4)+
259.2820
(M+H)+
260.2857
(M+H)+
Counts vs. Mass-to-Charge (m/z)

15

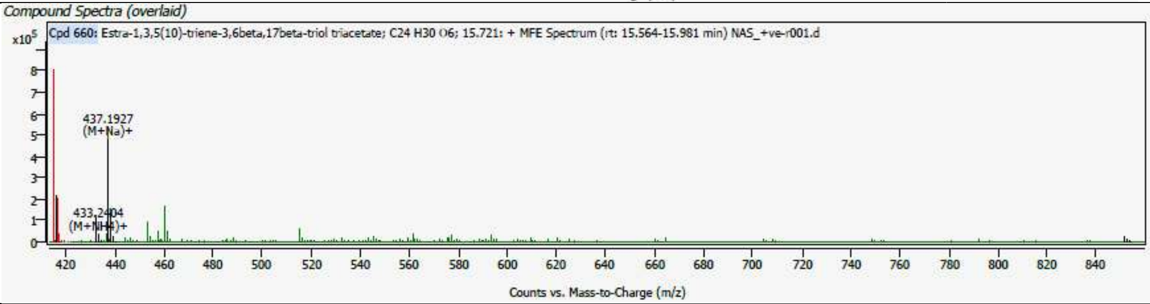
Compound Spectra (overlaid)
Cpd 660: Estra-1,3,5(10)-triene-3,6beta,17beta-triol triacetate; C24 H30 O6; 15.721: + MFE Spectrum (rt: 15.564-15.981 min) NAS_+ve-r001.d
x10^5
437.1927
(M+Na)+
433.2404
(M+NH4)+
Counts vs. Mass-to-Charge (m/z)

16

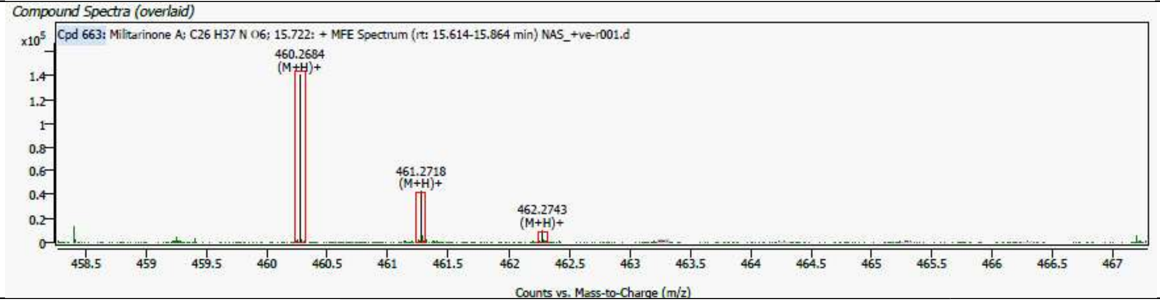
Compound Spectra (overlaid)
Cpd 663: Militarinone A; C26 H37 N O6; 15.722: + MFE Spectrum (rt: 15.614-15.864 min) NAS_+ve-r001.d
x10^5
460.2684
(M+H)+
461.2718
(M+H)+
462.2743
(M+H)+
Counts vs. Mass-to-Charge (m/z)

17

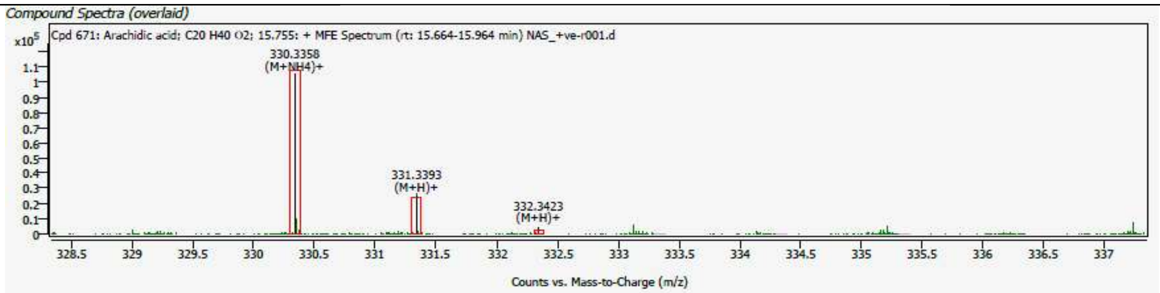
Compound Spectra (overlaid)
Cpd 671: Arachidic acid; C20 H40 O2; 15.755: + MFE Spectrum (rt: 15.664-15.964 min) NAS_+ve-r001.d
x10^5
330.3358 (M+NH4)+
331.3393 (M+H)+
332.3423 (M+H)+
Counts vs. Mass-to-Charge (m/z)

18

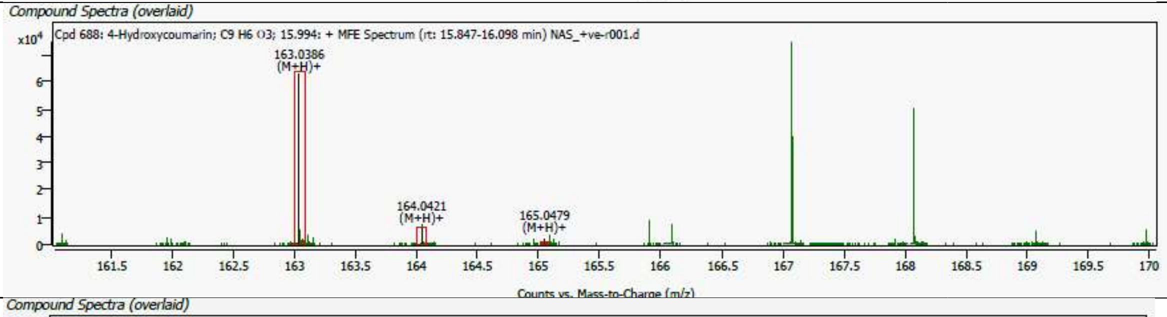
Compound Spectra (overlaid)
Cpd 688: 4-Hydroxycoumarin; C9 H6 O3; 15.994: + MFE Spectrum (rt: 15.847-16.098 min) NAS_+ve-r001.d
x10^4
163.0386 (M+H)+
164.0421 (M+H)+
165.0479 (M+H)+
Counts vs. Mass-to-Charge (m/z)

19

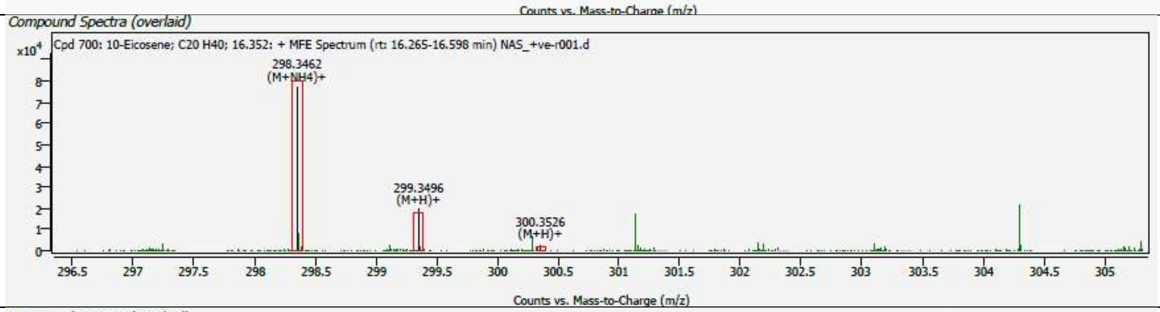
Compound Spectra (overlaid)
Cpd 700: 10-Eicosene; C20 H40; 16.352: + MFE Spectrum (rt: 16.265-16.598 min) NAS_+ve-r001.d
x10^4
298.3462 (M+NH4)+
299.3496 (M+H)+
300.3526 (M+H)+
Counts vs. Mass-to-Charge (m/z)

20

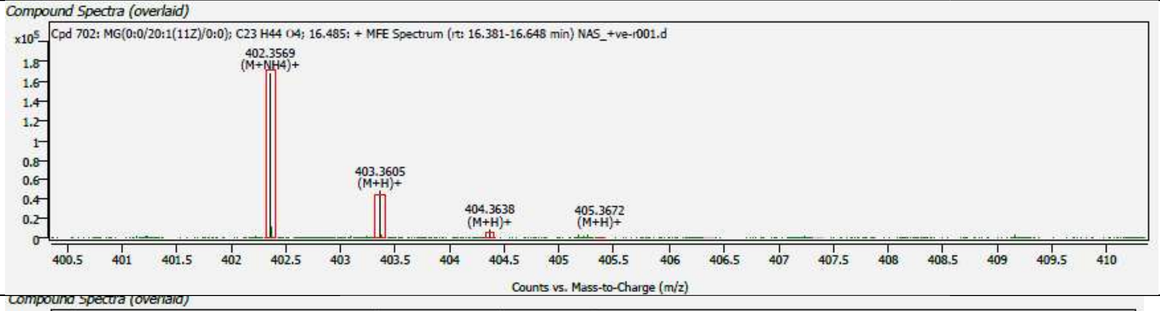
Compound Spectra (overlaid)
Cpd 702: MG(0:0/20:1(11Z)/0:0); C23 H44 O4; 16.485: + MFE Spectrum (rt: 16.381-16.648 min) NAS_+ve-r001.d
x10^5
402.3569 (M+NH4)+
403.3605 (M+H)+
404.3638 (M+H)+
405.3672 (M+H)+
Counts vs. Mass-to-Charge (m/z)

21

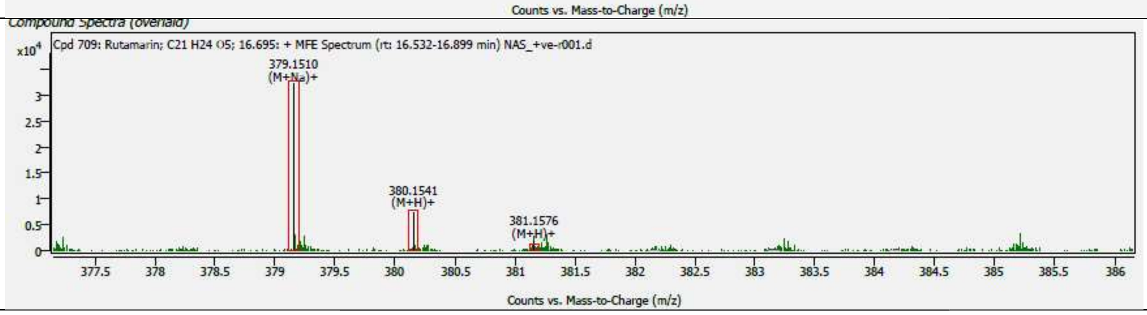
Compound Spectra (overlaid)
Cpd 709: Rutamarin; C21 H24 O5; 16.695: + MFE Spectrum (rt: 16.532-16.899 min) NAS_+ve-r001.d
x10^4
379.1510 (M+Na)+
380.1541 (M+H)+
381.1576 (M+H)+
Counts vs. Mass-to-Charge (m/z)

22

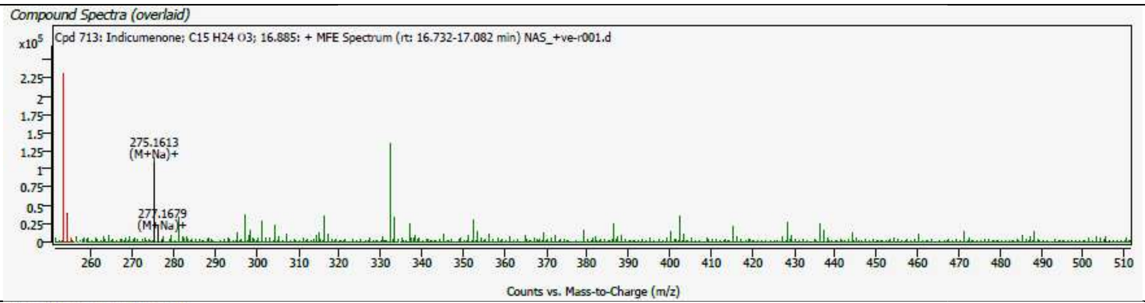
Compound Spectra (overlaid)
Cpd 713: Indicumenone; C15 H24 O3; 16.885: + MFE Spectrum (rt: 16.732-17.082 min) NAS_+ve-r001.d
x10^5
275.1613
(M+Na)+
277.1679
(M+Na)+
Counts vs. Mass-to-Charge (m/z)

23

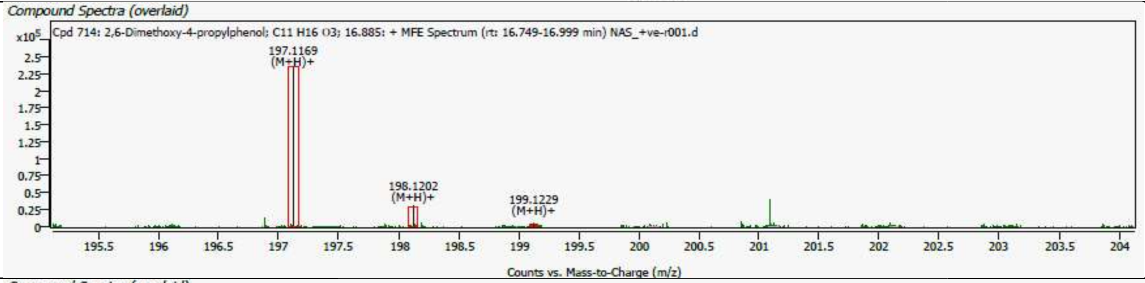
Compound Spectra (overlaid)
Cpd 714: 2,6-Dimethoxy-4-propylphenol; C11 H16 O3; 16.885: + MFE Spectrum (rt: 16.749-16.999 min) NAS_+ve-r001.d
x10^5
197.1169
(M+H)+
198.1202
(M+H)+
199.1229
(M+H)+
Counts vs. Mass-to-Charge (m/z)

24

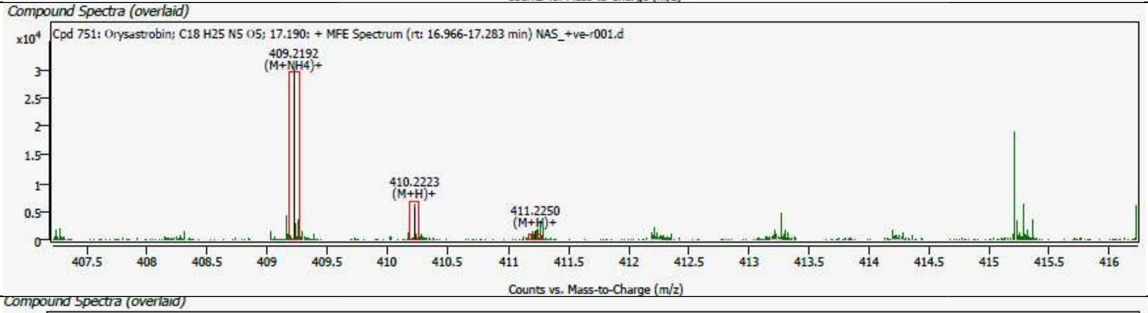
Compound Spectra (overlaid)
Cpd 751: Orysastrobin; C18 H25 N5 O5; 17.190: + MFE Spectrum (rt: 16.966-17.283 min) NAS_+ve-r001.d
x10^4
409.2192
(M+NH4)+
410.2223
(M+H)+
411.2250
(M+H)+
Counts vs. Mass-to-Charge (m/z)

25

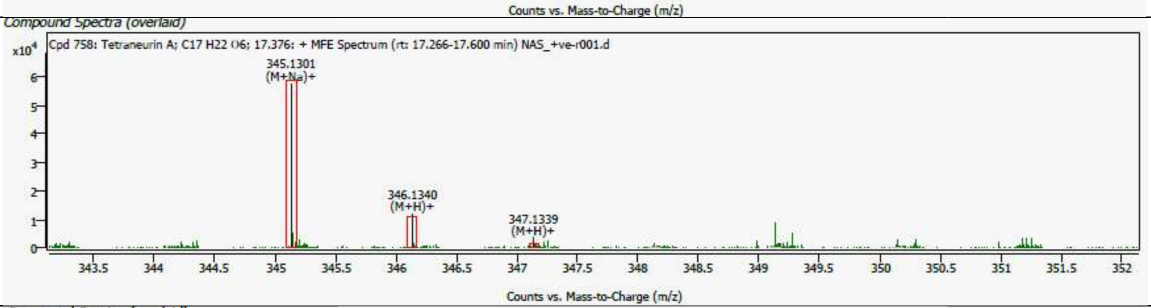
Compound Spectra (overlaid)
Cpd 758: Tetraneurin A; C17 H22 O6; 17.376: + MFE Spectrum (rt: 17.266-17.600 min) NAS_+ve-r001.d
x10^4
345.1301
(M+Na)+
346.1340
(M+H)+
347.1339
(M+H)+
Counts vs. Mass-to-Charge (m/z)

26

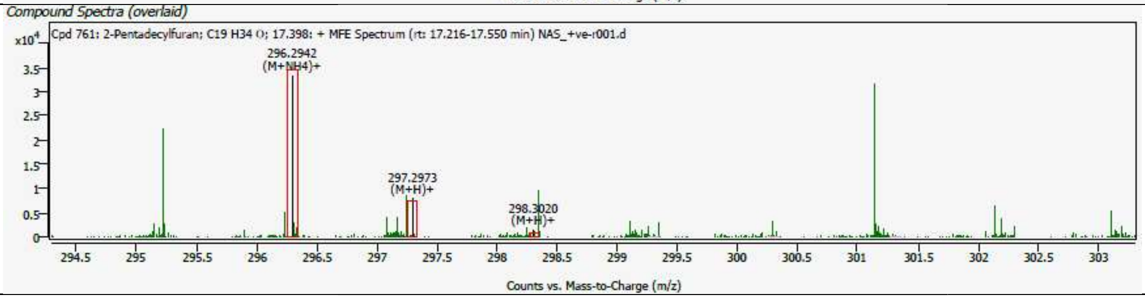
Compound Spectra (overlaid)
Cpd 761: 2-Pentadecylfuran; C19 H34 O; 17.398: + MFE Spectrum (rt: 17.216-17.550 min) NAS_+ve-r001.d
x10^4
296.2942
(M+NH4)+
297.2973
(M+H)+
298.3020
(M+H)+
Counts vs. Mass-to-Charge (m/z)

27

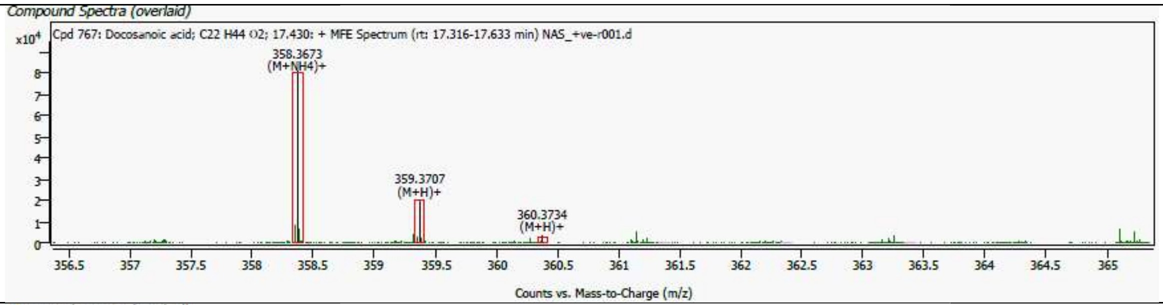
Compound Spectra (overlaid)
x10^4
Cpd 767: Docosanoic acid; C22 H44 O2; 17.430: + MFE Spectrum (rt: 17.316-17.633 min) NAS_+ve-r001.d
358.3673 (M+NH4)+
359.3707 (M+H)+
360.3734 (M+H)+
Counts vs. Mass-to-Charge (m/z)

28

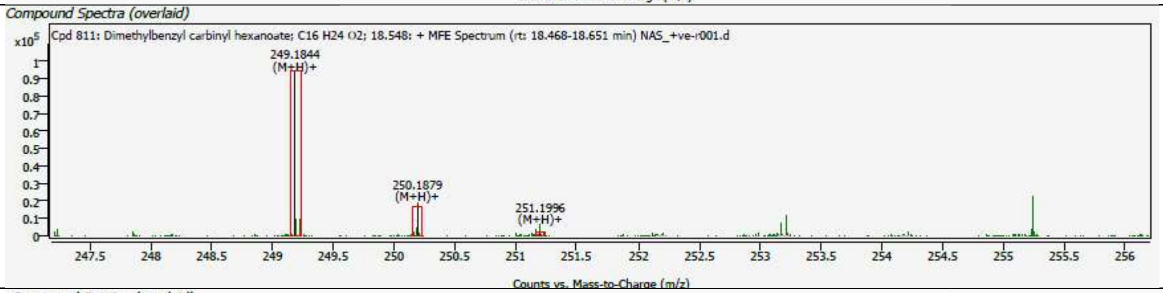
Compound Spectra (overlaid)
x10^5
Cpd 811: Dimethylbenzyl carbinyl hexanoate; C16 H24 O2; 18.548: + MFE Spectrum (rt: 18.468-18.651 min) NAS_+ve-r001.d
249.1844 (M+H)+
250.1879 (M+H)+
251.1996 (M+H)+
Counts vs. Mass-to-Charge (m/z)

29

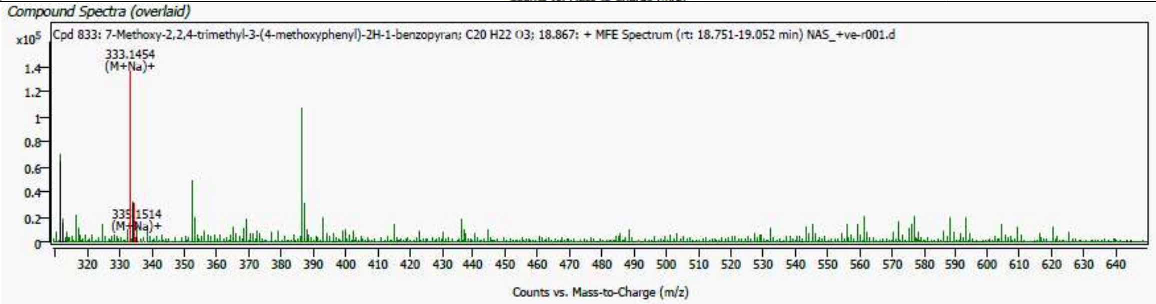
Compound Spectra (overlaid)
x10^5
Cpd 833: 7-Methoxy-2,2,4-trimethyl-3-(4-methoxyphenyl)-2H-1-benzopyran; C20 H22 O3; 18.867: + MFE Spectrum (rt: 18.751-19.052 min) NAS_+ve-r001.d
333.1454 (M+Na)+
335.1514 (M+Na)+
Counts vs. Mass-to-Charge (m/z)

30

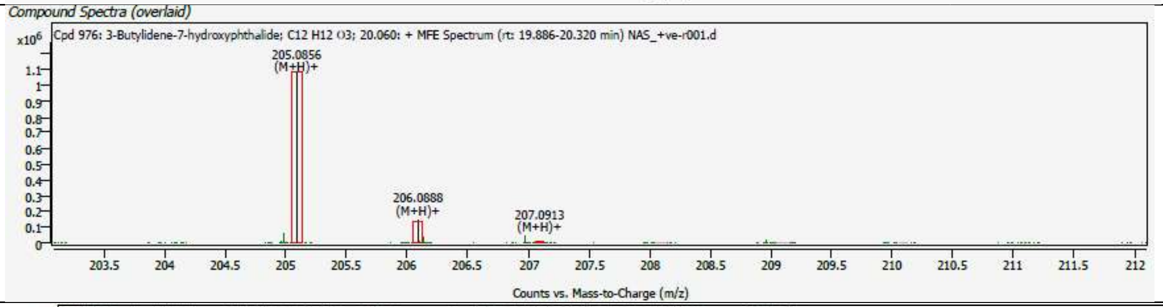
Compound Spectra (overlaid)
x10^6
Cpd 976: 3-Butylidene-7-hydroxyphthalide; C12 H12 O3; 20.060: + MFE Spectrum (rt: 19.886-20.320 min) NAS_+ve-r001.d
205.0856 (M+H)+
206.0888 (M+H)+
207.0913 (M+H)+
Counts vs. Mass-to-Charge (m/z)

31

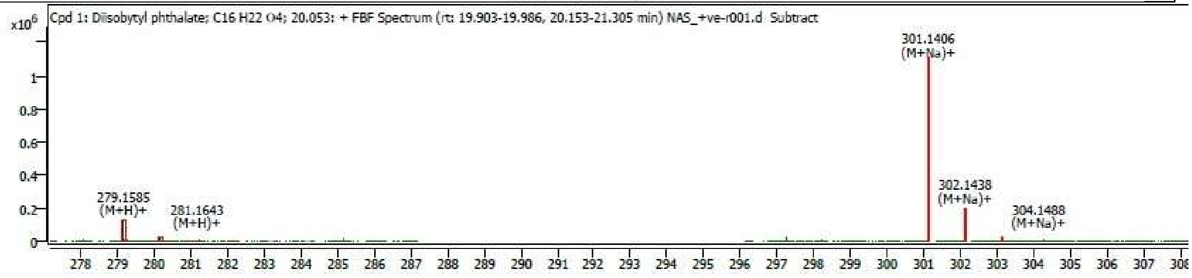
x10^6
Cpd 1: Diisobutyl phthalate; C16 H22 O4; 20.053: + FBF Spectrum (rt: 19.903-19.986, 20.153-21.305 min) NAS_+ve-r001.d Subtract
301.1406 (M+Na)+
302.1438 (M+Na)+
304.1488 (M+Na)+
279.1585 (M+H)+
281.1643 (M+H)+

32	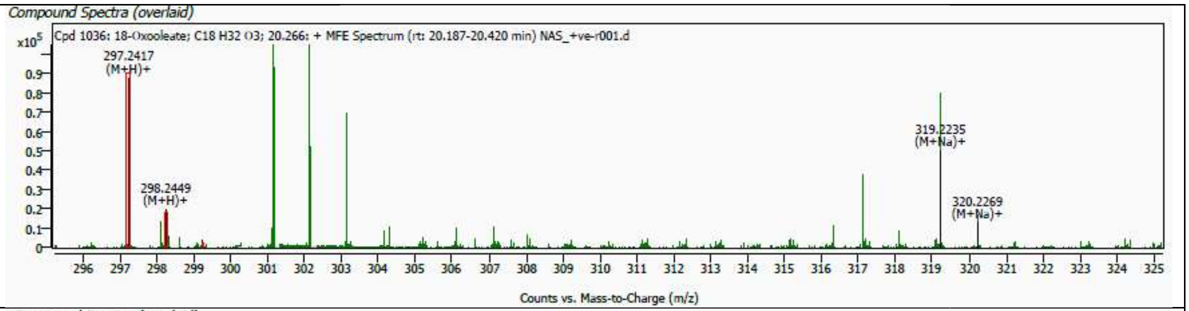Compound Spectra (overlaid)<br>Cpd 1036: 18-Oxooleate; C18 H32 O3; 20.266: + MFE Spectrum (rt: 20.187-20.420 min) NAS_+ve-r001.d<br>297.2417 (M+H)+<br>298.2449 (M+H)+<br>319.2235 (M+Na)+<br>320.2269 (M+Na)+<br>Counts vs. Mass-to-Charge (m/z)
33	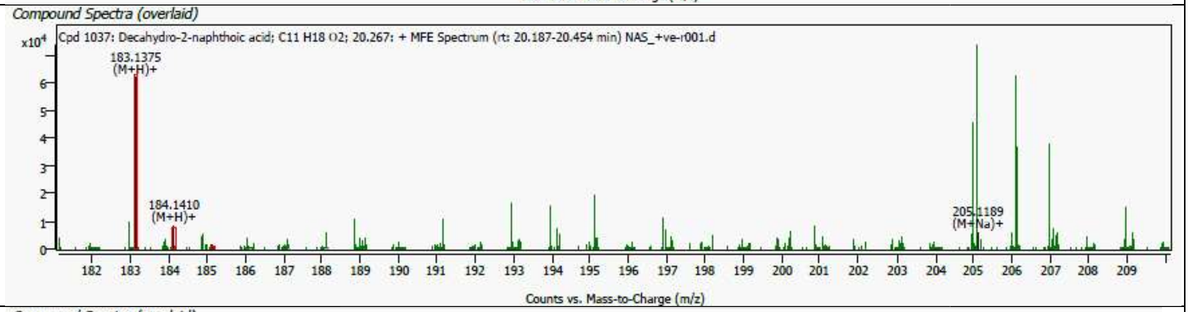Compound Spectra (overlaid)<br>Cpd 1037: Decahydro-2-naphthoic acid; C11 H18 O2; 20.267: + MFE Spectrum (rt: 20.187-20.454 min) NAS_+ve-r001.d<br>183.1375 (M+H)+<br>184.1410 (M+H)+<br>205.1189 (M+Na)+<br>Counts vs. Mass-to-Charge (m/z)
34	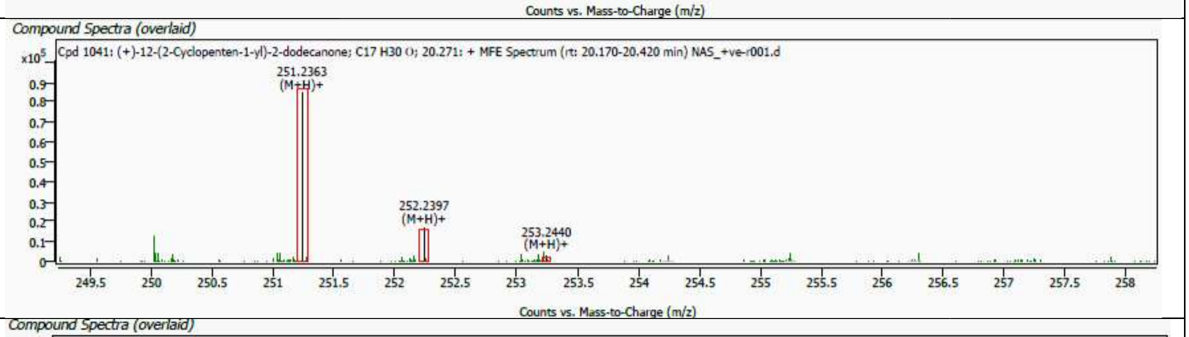Compound Spectra (overlaid)<br>Cpd 1041: (+)-12-(2-Cyclopenten-1-yl)-2-dodecanone; C17 H30 O; 20.271: + MFE Spectrum (rt: 20.170-20.420 min) NAS_+ve-r001.d<br>251.2363 (M+H)+<br>252.2397 (M+H)+<br>253.2440 (M+H)+<br>Counts vs. Mass-to-Charge (m/z)
35	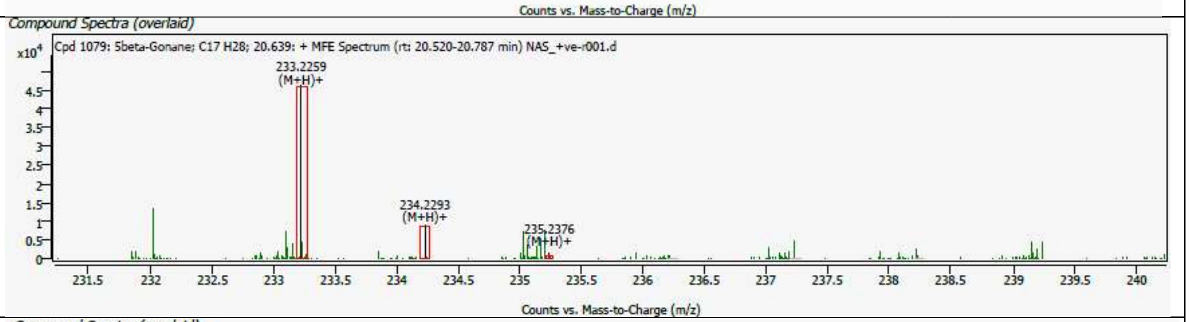Compound Spectra (overlaid)<br>Cpd 1079: 5beta-Gonane; C17 H28; 20.639: + MFE Spectrum (rt: 20.520-20.787 min) NAS_+ve-r001.d<br>233.2259 (M+H)+<br>234.2293 (M+H)+<br>235.2376 (M+H)+<br>Counts vs. Mass-to-Charge (m/z)
36	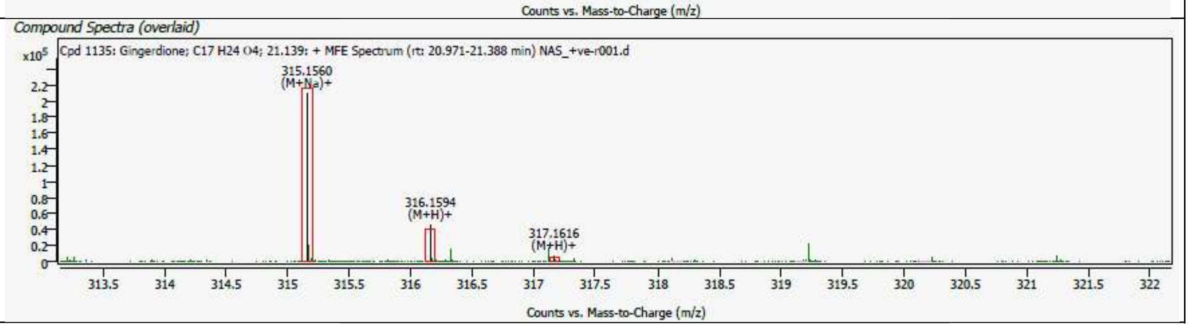Compound Spectra (overlaid)<br>Cpd 1135: Gingerdione; C17 H24 O4; 21.139: + MFE Spectrum (rt: 20.971-21.388 min) NAS_+ve-r001.d<br>315.1560 (M+Na)+<br>316.1594 (M+H)+<br>317.1616 (M+H)+<br>Counts vs. Mass-to-Charge (m/z)

37

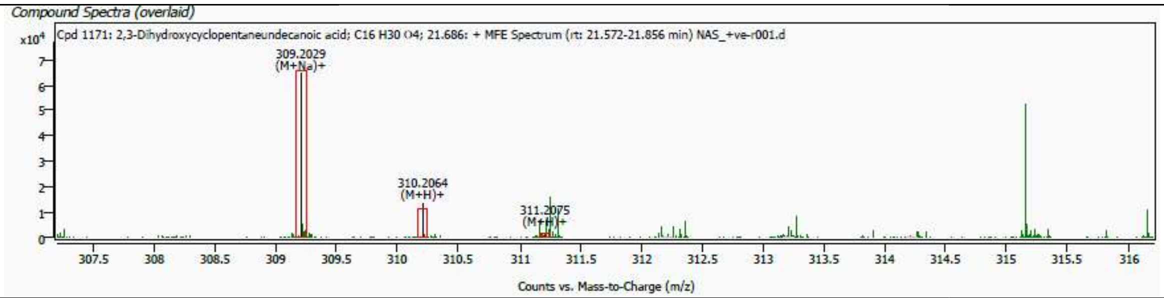
Compound Spectra (overlaid)
Cpd 1171: 2,3-Dihydroxycyclopentaneundecanoic acid; C16 H30 O4; 21.686: + MFE Spectrum (rt: 21.572-21.856 min) NAS_+ve-r001.d
x10^4
309.2029
(M+Na)+
310.2064
(M+H)+
311.2075
(M+H)+
Counts vs. Mass-to-Charge (m/z)

38

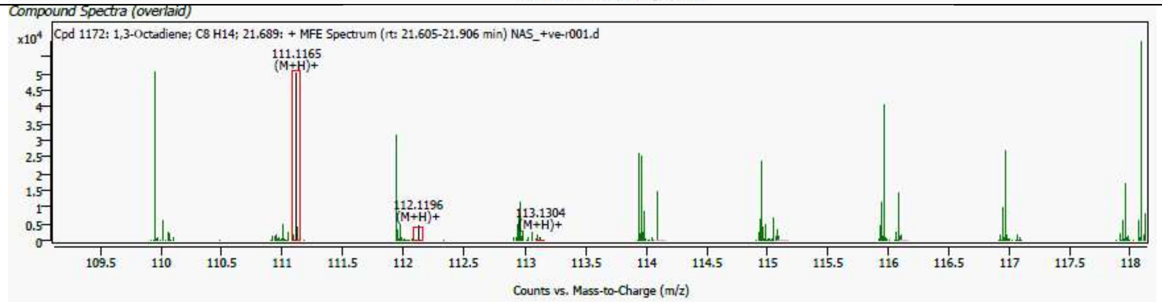
Compound Spectra (overlaid)
Cpd 1172: 1,3-Octadiene; C8 H14; 21.689: + MFE Spectrum (rt: 21.605-21.906 min) NAS_+ve-r001.d
x10^4
111.1165
(M+H)+
112.1196
(M+H)+
113.1304
(M+H)+
Counts vs. Mass-to-Charge (m/z)

39

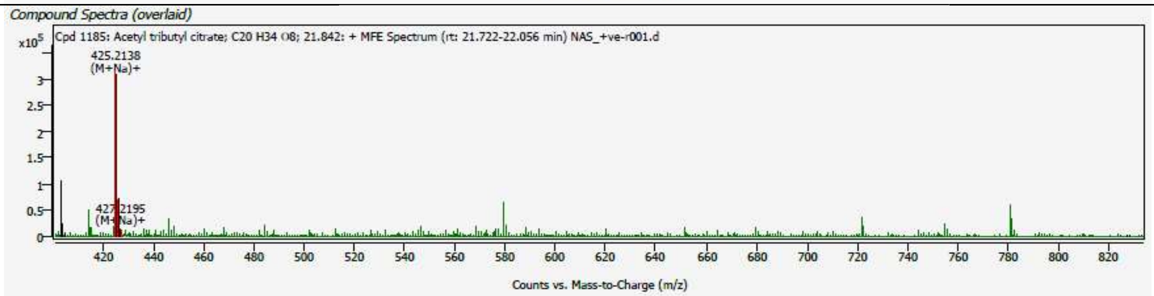
Compound Spectra (overlaid)
Cpd 1185: Acetyl tributyl citrate; C20 H34 O8; 21.842: + MFE Spectrum (rt: 21.722-22.056 min) NAS_+ve-r001.d
x10^5
425.2138
(M+Na)+
427.2195
(M+Na)+
Counts vs. Mass-to-Charge (m/z)

40

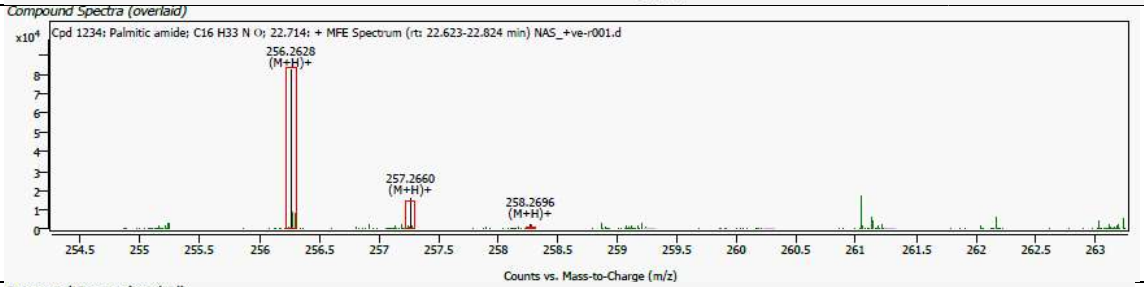
Compound Spectra (overlaid)
Cpd 1234: Palmitic amide; C16 H33 N O; 22.714: + MFE Spectrum (rt: 22.623-22.824 min) NAS_+ve-r001.d
x10^4
256.2628
(M+H)+
257.2660
(M+H)+
258.2696
(M+H)+
Counts vs. Mass-to-Charge (m/z)

41

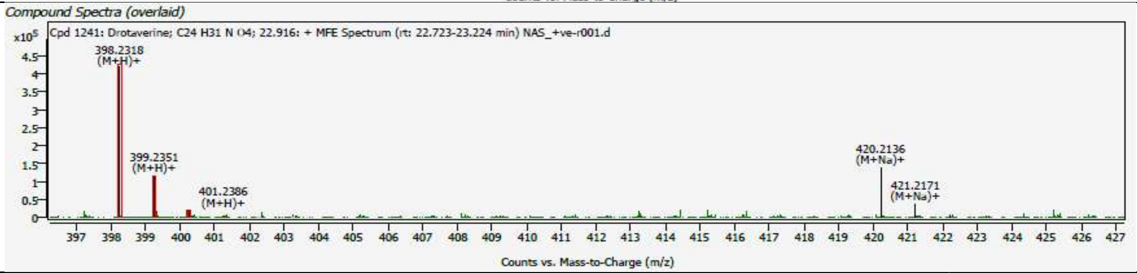
Compound Spectra (overlaid)
Cpd 1241: Drotaverine; C24 H31 N O4; 22.916: + MFE Spectrum (rt: 22.723-23.224 min) NAS_+ve-r001.d
x10^5
398.2318
(M+H)+
399.2351
(M+H)+
401.2386
(M+H)+
420.2136
(M+Na)+
421.2171
(M+Na)+
Counts vs. Mass-to-Charge (m/z)

42	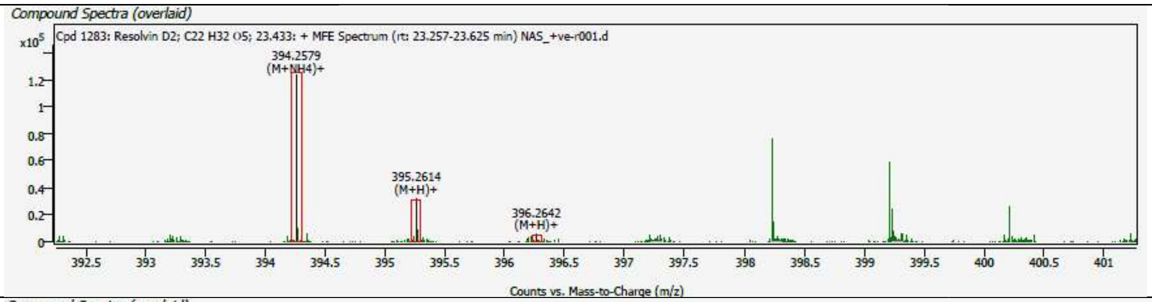Compound Spectra (overlaid)<br>Cpd 1283: Resolvin D2; C22 H32 O5; 23.433: + MFE Spectrum (rt: 23.257-23.625 min) NAS_+ve-r001.d<br>394.2579 (M+NH4)+<br>395.2614 (M+H)+<br>396.2642 (M+H)+<br>Counts vs. Mass-to-Charge (m/z)
43	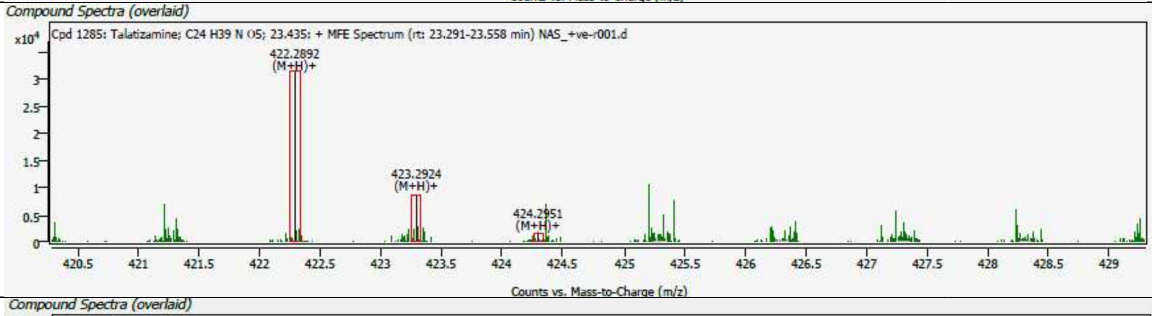Compound Spectra (overlaid)<br>Cpd 1285: Talatizamine; C24 H39 N O5; 23.435: + MFE Spectrum (rt: 23.291-23.558 min) NAS_+ve-r001.d<br>422.2892 (M+H)+<br>423.2924 (M+H)+<br>424.2951 (M+H)+<br>Counts vs. Mass-to-Charge (m/z)
44	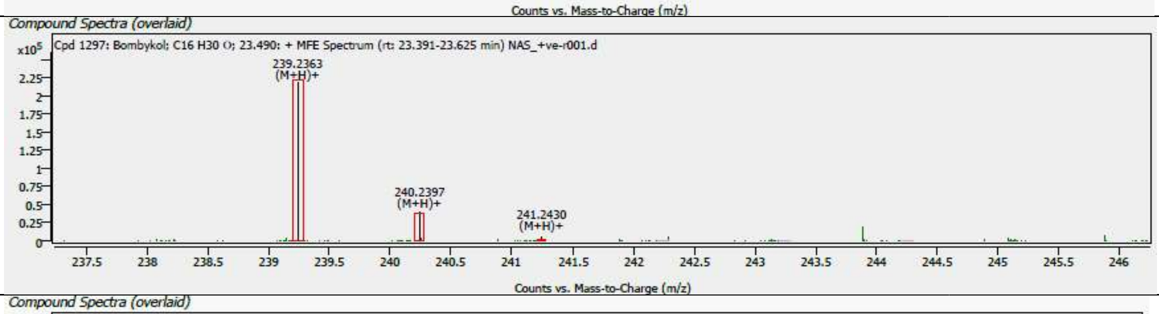Compound Spectra (overlaid)<br>Cpd 1297: Bombykol; C16 H30 O; 23.490: + MFE Spectrum (rt: 23.391-23.625 min) NAS_+ve-r001.d<br>239.2363 (M+H)+<br>240.2397 (M+H)+<br>241.2430 (M+H)+<br>Counts vs. Mass-to-Charge (m/z)
45	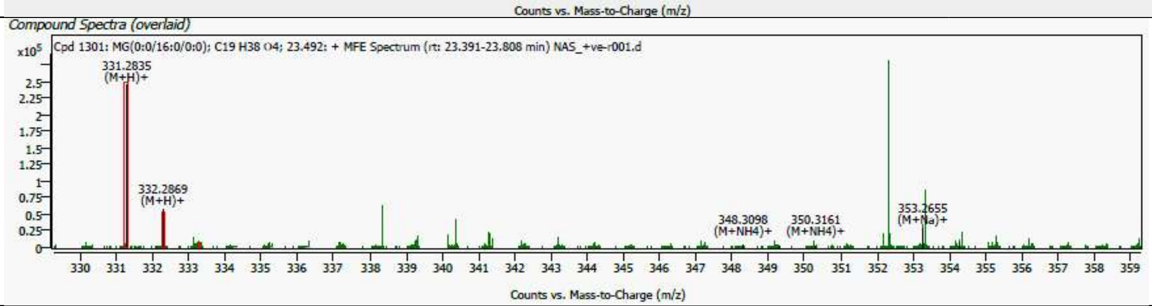Compound Spectra (overlaid)<br>Cpd 1301: MG(0:0/16:0/0:0); C19 H38 O4; 23.492: + MFE Spectrum (rt: 23.391-23.808 min) NAS_+ve-r001.d<br>331.2835 (M+H)+<br>332.2869 (M+H)+<br>348.3098 (M+NH4)+<br>350.3161 (M+NH4)+<br>353.2655 (M+Na)+<br>Counts vs. Mass-to-Charge (m/z)
46	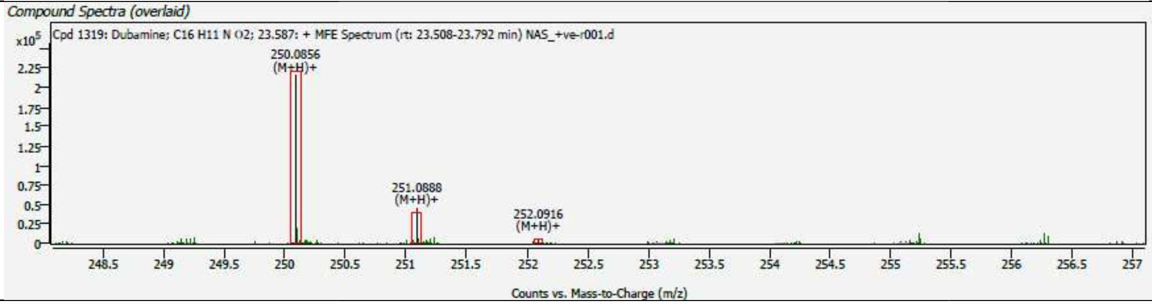Compound Spectra (overlaid)<br>Cpd 1319: Dubamine; C16 H11 N O2; 23.587: + MFE Spectrum (rt: 23.508-23.792 min) NAS_+ve-r001.d<br>250.0856 (M+H)+<br>251.0888 (M+H)+<br>252.0916 (M+H)+<br>Counts vs. Mass-to-Charge (m/z)

47	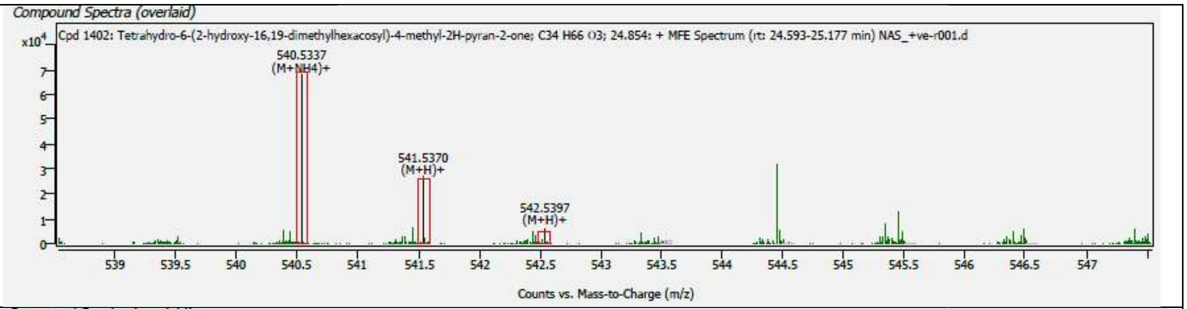
48	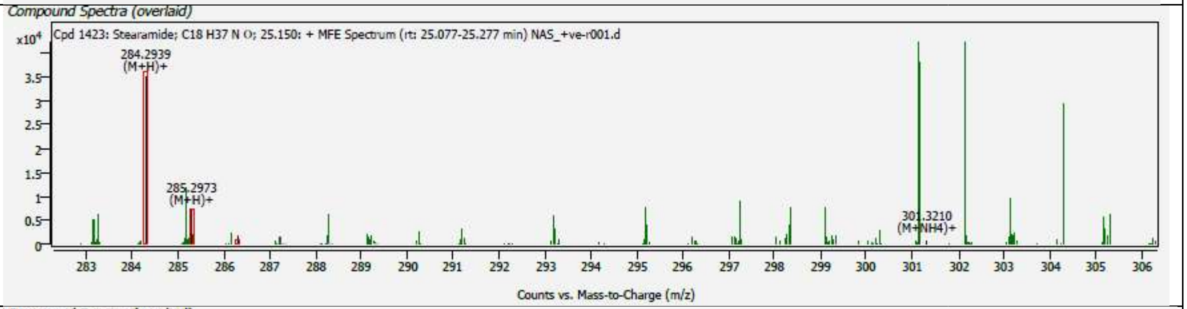
49	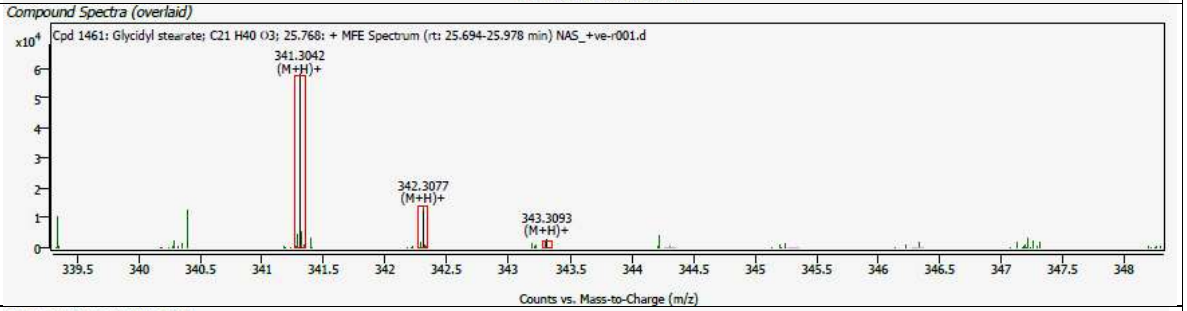
50	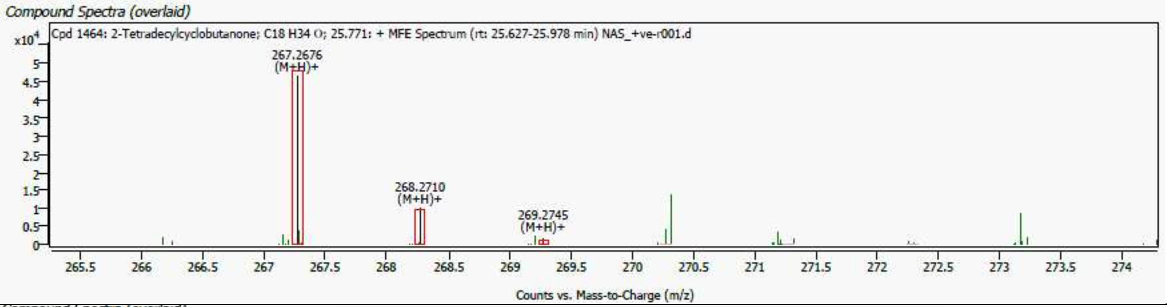
51	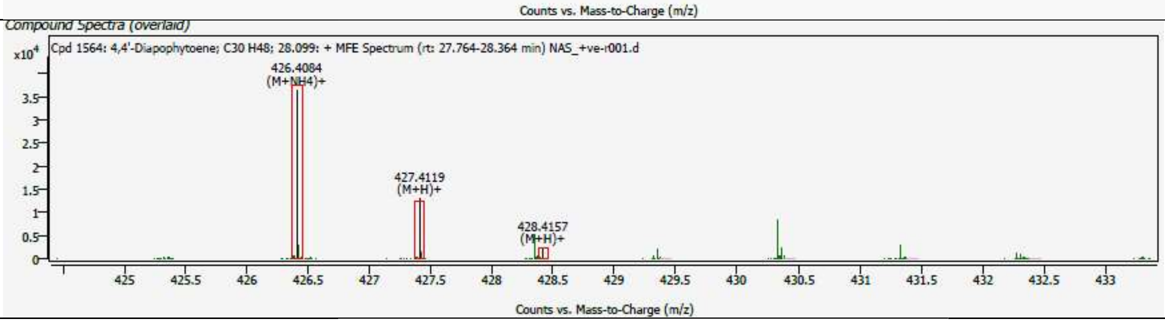

Compound Spectra (overlaid)
Cpd 1402: Tetrahydro-6-(2-hydroxy-16,19-dimethylhexacosyl)-4-methyl-2H-pyran-2-one; C34 H66 O3; 24.854: + MFE Spectrum (rt: 24.593-25.177 min) NAS_+ve-r001.d
540.5337 (M+NH4)+
541.5370 (M+H)+
542.5397 (M+H)+
Counts vs. Mass-to-Charge (m/z)

Compound Spectra (overlaid)
Cpd 1423: Stearamide; C18 H37 N O; 25.150: + MFE Spectrum (rt: 25.077-25.277 min) NAS_+ve-r001.d
284.2939 (M+H)+
285.2973 (M+H)+
301.3210 (M+NH4)+
Counts vs. Mass-to-Charge (m/z)

Compound Spectra (overlaid)
Cpd 1461: Glycidyl stearate; C21 H40 O3; 25.768: + MFE Spectrum (rt: 25.694-25.978 min) NAS_+ve-r001.d
341.3042 (M+H)+
342.3077 (M+H)+
343.3093 (M+H)+
Counts vs. Mass-to-Charge (m/z)

Compound Spectra (overlaid)
Cpd 1464: 2-Tetradecylcyclobutanone; C18 H34 O; 25.771: + MFE Spectrum (rt: 25.627-25.978 min) NAS_+ve-r001.d
267.2676 (M+H)+
268.2710 (M+H)+
269.2745 (M+H)+
Counts vs. Mass-to-Charge (m/z)

Compound Spectra (overlaid)
Cpd 1564: 4,4'-Diapophytoene; C30 H48; 28.099: + MFE Spectrum (rt: 27.764-28.364 min) NAS_+ve-r001.d
426.4084 (M+NH4)+
427.4119 (M+H)+
428.4157 (M+H)+
Counts vs. Mass-to-Charge (m/z)

52	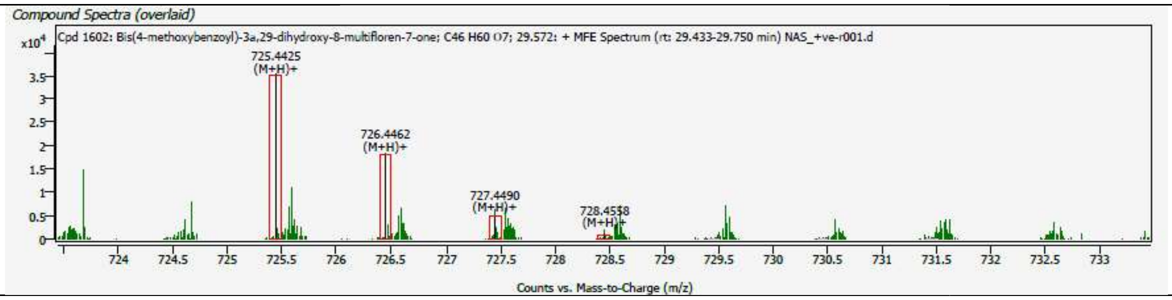Compound Spectra (overlaid)<br>Cpd 1602: Bis(4-methoxybenzoyl)-3a,29-dihydroxy-8-multifloren-7-one; C46 H60 O7; 29.572: + MFE Spectrum (rt: 29.433-29.750 min) NAS_+ve-r001.d<br>725.4425 (M+H)+<br>726.4462 (M+H)+<br>727.4490 (M+H)+<br>728.4558 (M+H)+<br>Counts vs. Mass-to-Charge (m/z)
53	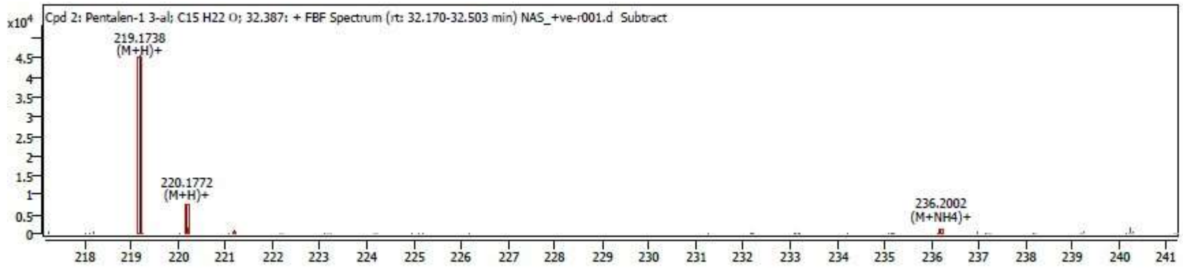Cpd 2: Pentalen-1 3-al; C15 H22 O; 32.387: + FBF Spectrum (rt: 32.170-32.503 min) NAS_+ve-r001.d Subtract<br>219.1738 (M+H)+<br>220.1772 (M+H)+<br>236.2002 (M+NH4)+
54	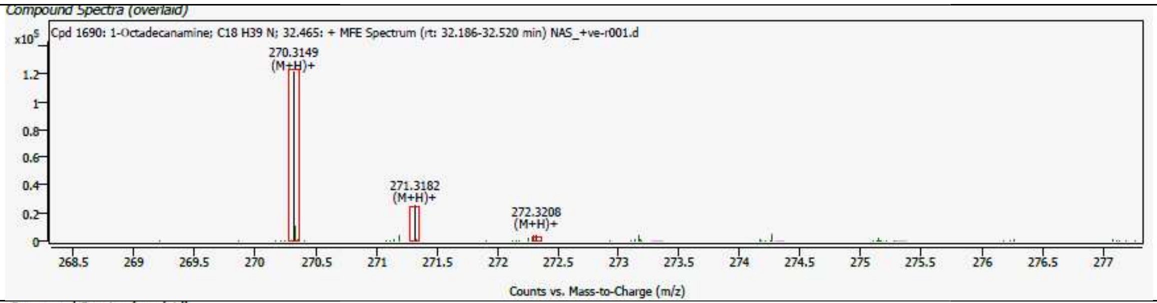Compound Spectra (overlaid)<br>Cpd 1690: 1-Octadecanamine; C18 H39 N; 32.465: + MFE Spectrum (rt: 32.186-32.520 min) NAS_+ve-r001.d<br>270.3149 (M+H)+<br>271.3182 (M+H)+<br>272.3208 (M+H)+<br>Counts vs. Mass-to-Charge (m/z)
55	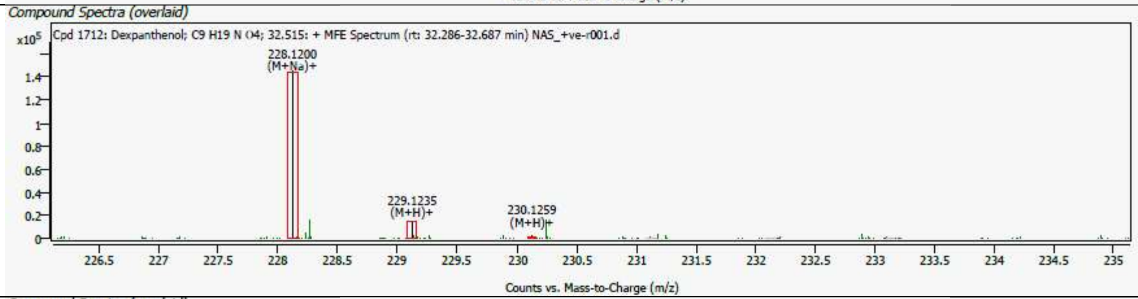Compound Spectra (overlaid)<br>Cpd 1712: Dexpanthenol; C9 H19 N O4; 32.515: + MFE Spectrum (rt: 32.286-32.687 min) NAS_+ve-r001.d<br>228.1200 (M+Na)+<br>229.1235 (M+H)+<br>230.1259 (M+H)+<br>Counts vs. Mass-to-Charge (m/z)
56	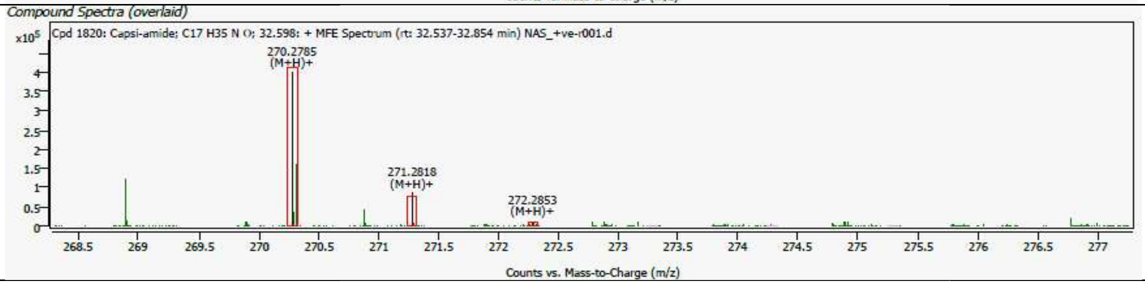Compound Spectra (overlaid)<br>Cpd 1820: Capsi-amide; C17 H35 N O; 32.598: + MFE Spectrum (rt: 32.537-32.854 min) NAS_+ve-r001.d<br>270.2785 (M+H)+<br>271.2818 (M+H)+<br>272.2853 (M+H)+<br>Counts vs. Mass-to-Charge (m/z)

<table>
<tr><th>Cpd.</th><th>Figure S2. UPLC–HRMS Spectra of compounds indentified by negative ionization mode</th></tr>
<tr><td>57</td><td>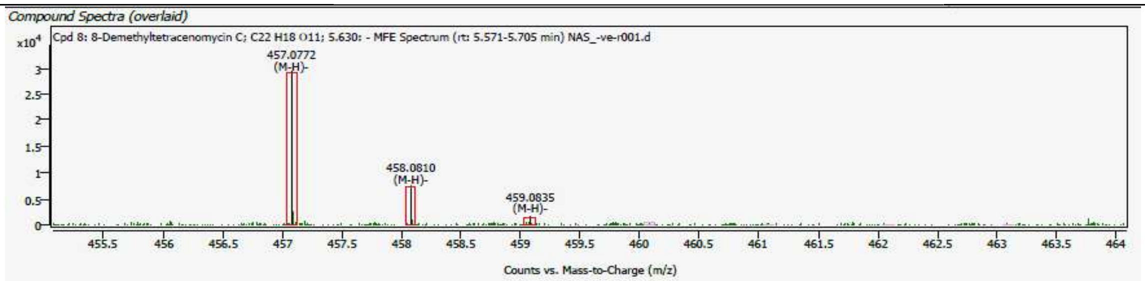
</td></tr>
<tr><td>58</td><td>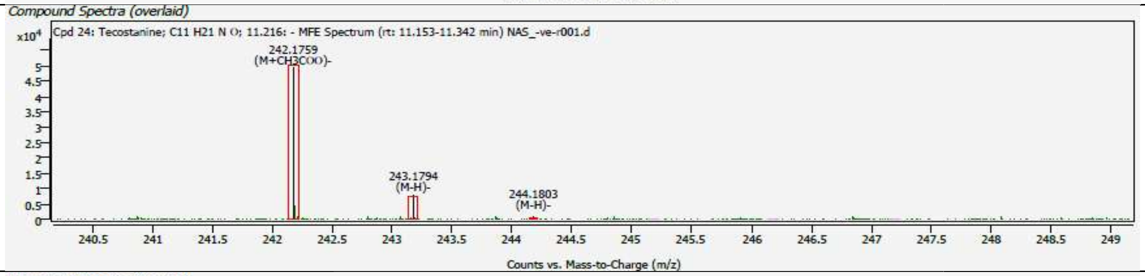
</td></tr>
<tr><td>59</td><td>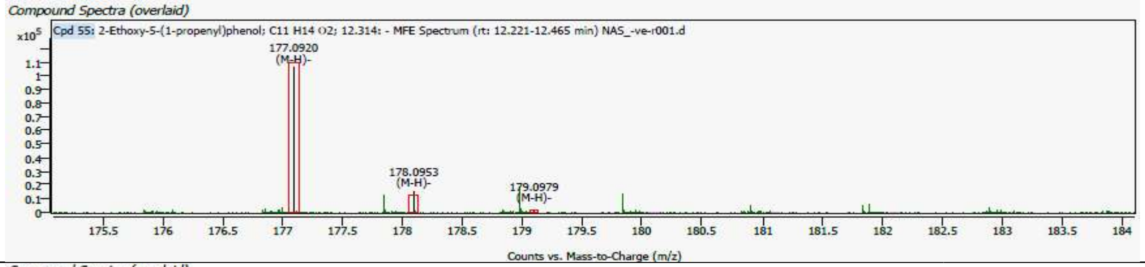
</td></tr>
<tr><td>60</td><td>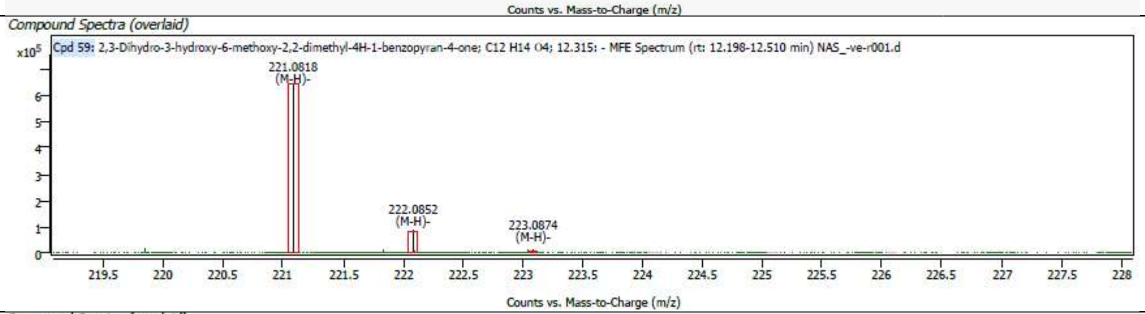
</td></tr>
<tr><td>61</td><td>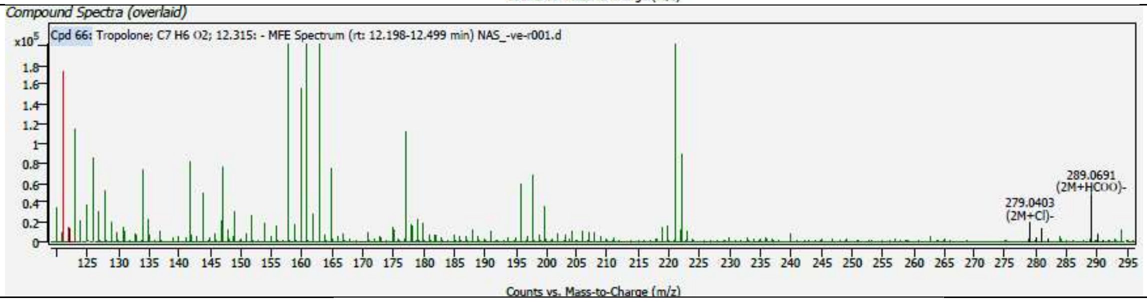
</td></tr>
</table>

62

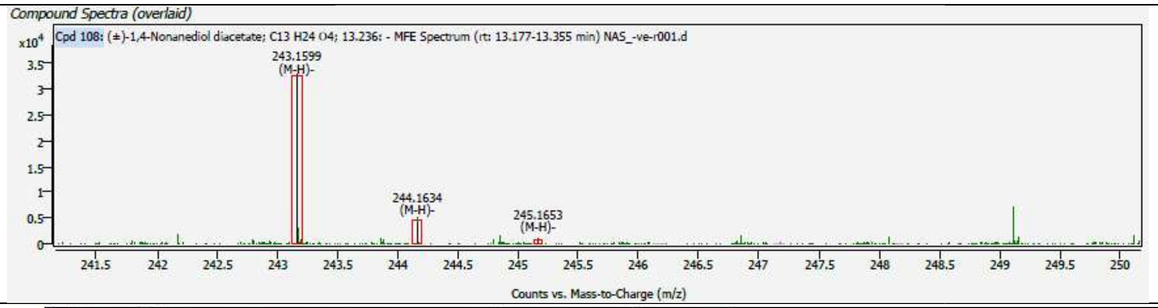
Compound Spectra (overlaid)
Cpd 108: (±)-1,4-Nonanediol diacetate; C13 H24 O4; 13.236: - MFE Spectrum (rt: 13.177-13.355 min) NAS_-ve-r001.d
x10^4
243.1599
(M-H)-
244.1634
(M-H)-
245.1653
(M-H)-
Counts vs. Mass-to-Charge (m/z)

63

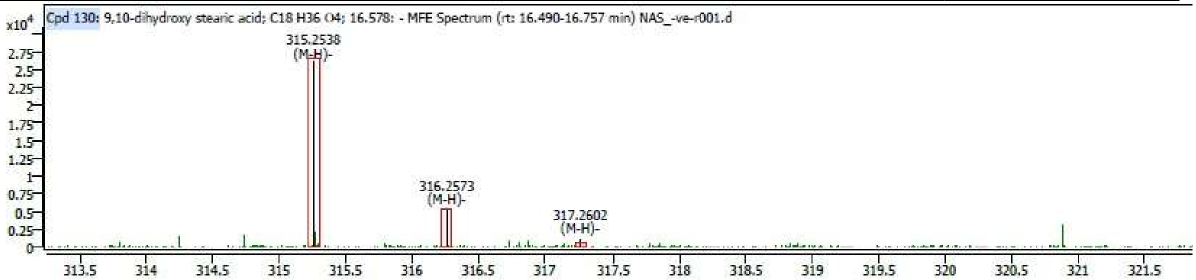
Cpd 130: 9,10-dihydroxy stearic acid; C18 H36 O4; 16.578: - MFE Spectrum (rt: 16.490-16.757 min) NAS_-ve-r001.d
x10^4
315.2538
(M-H)-
316.2573
(M-H)-
317.2602
(M-H)-

64

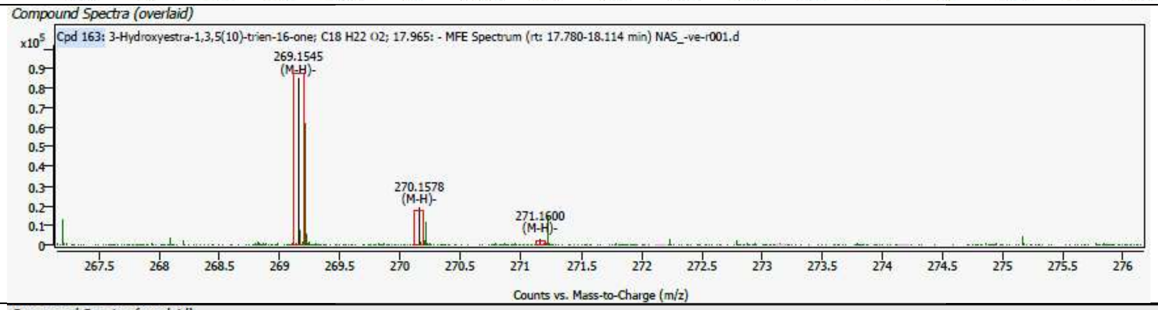
Compound Spectra (overlaid)
Cpd 163: 3-Hydroxyestra-1,3,5(10)-trien-16-one; C18 H22 O2; 17.965: - MFE Spectrum (rt: 17.780-18.114 min) NAS_-ve-r001.d
x10^5
269.1545
(M-H)-
270.1578
(M-H)-
271.1600
(M-H)-
Counts vs. Mass-to-Charge (m/z)

65

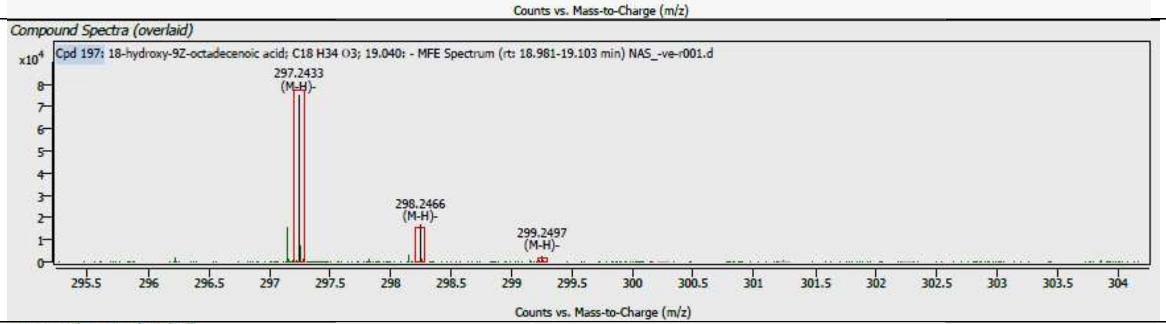
Compound Spectra (overlaid)
Cpd 197: 18-hydroxy-9Z-octadecenoic acid; C18 H34 O3; 19.040: - MFE Spectrum (rt: 18.981-19.103 min) NAS_-ve-r001.d
x10^4
297.2433
(M-H)-
298.2466
(M-H)-
299.2497
(M-H)-
Counts vs. Mass-to-Charge (m/z)

66

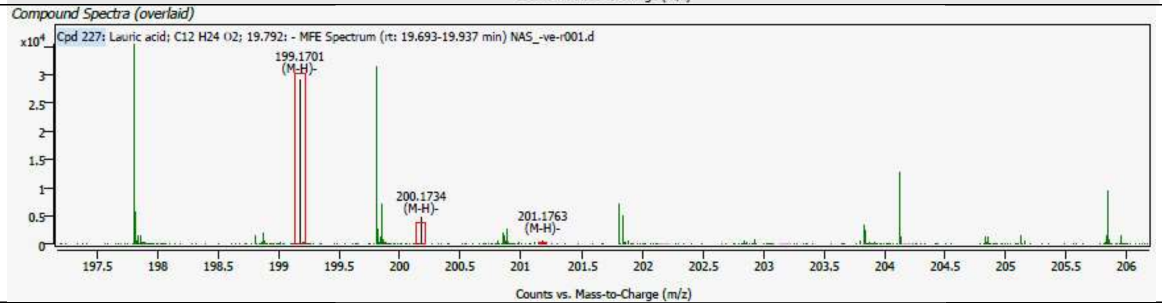
Compound Spectra (overlaid)
Cpd 227: Lauric acid; C12 H24 O2; 19.792: - MFE Spectrum (rt: 19.693-19.937 min) NAS_-ve-r001.d
x10^4
199.1701
(M-H)-
200.1734
(M-H)-
201.1763
(M-H)-
Counts vs. Mass-to-Charge (m/z)

67

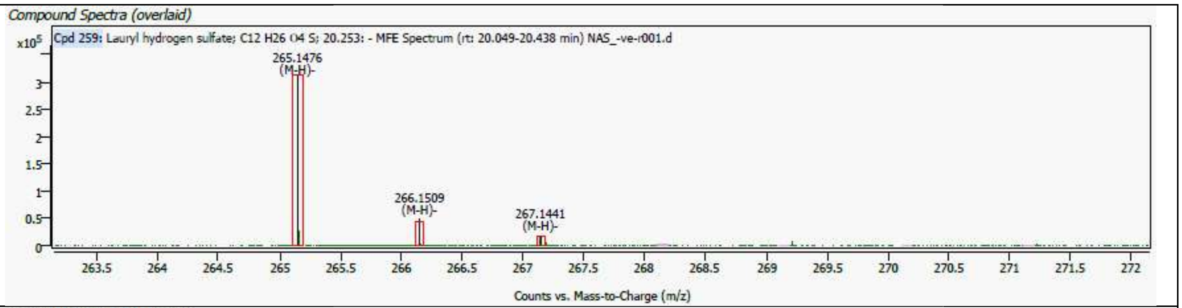
Compound Spectra (overlaid)
x10^5
Cpd 259: Lauryl hydrogen sulfate; C12 H26 O4 S; 20.253: - MFE Spectrum (rt: 20.049-20.438 min) NAS_-ve-r001.d
265.1476 (M-H)-
266.1509 (M-H)-
267.1441 (M-H)-
Counts vs. Mass-to-Charge (m/z)

68

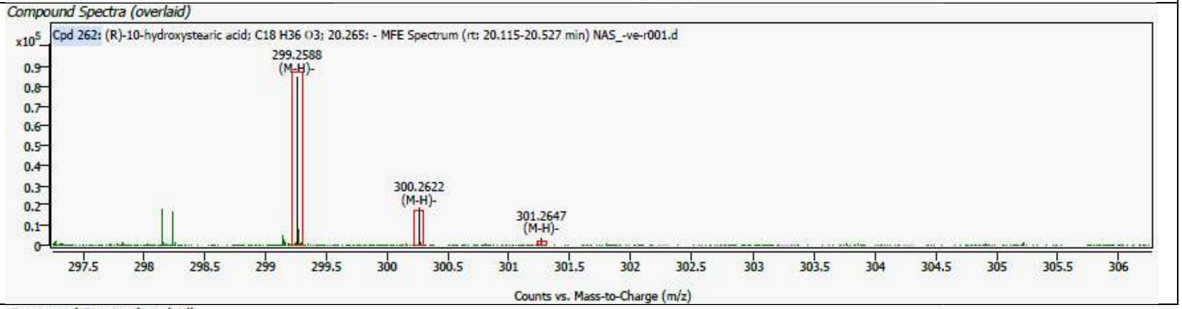
Compound Spectra (overlaid)
x10^5
Cpd 262: (R)-10-hydroxystearic acid; C18 H36 O3; 20.265: - MFE Spectrum (rt: 20.115-20.527 min) NAS_-ve-r001.d
299.2588 (M-H)-
300.2622 (M-H)-
301.2647 (M-H)-
Counts vs. Mass-to-Charge (m/z)

69

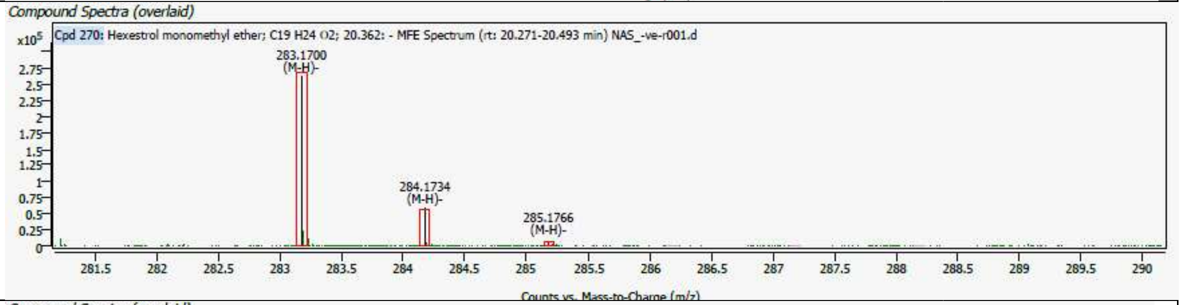
Compound Spectra (overlaid)
x10^5
Cpd 270: Hexestrol monomethyl ether; C19 H24 O2; 20.362: - MFE Spectrum (rt: 20.271-20.493 min) NAS_-ve-r001.d
283.1700 (M-H)-
284.1734 (M-H)-
285.1766 (M-H)-
Counts vs. Mass-to-Charge (m/z)

70

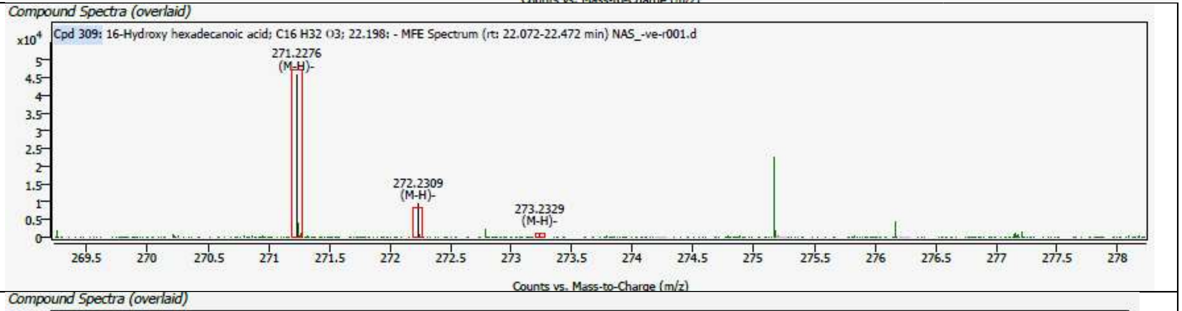
Compound Spectra (overlaid)
x10^4
Cpd 309: 16-Hydroxy hexadecanoic acid; C16 H32 O3; 22.198: - MFE Spectrum (rt: 22.072-22.472 min) NAS_-ve-r001.d
271.2276 (M-H)-
272.2309 (M-H)-
273.2329 (M-H)-
Counts vs. Mass-to-Charge (m/z)

71

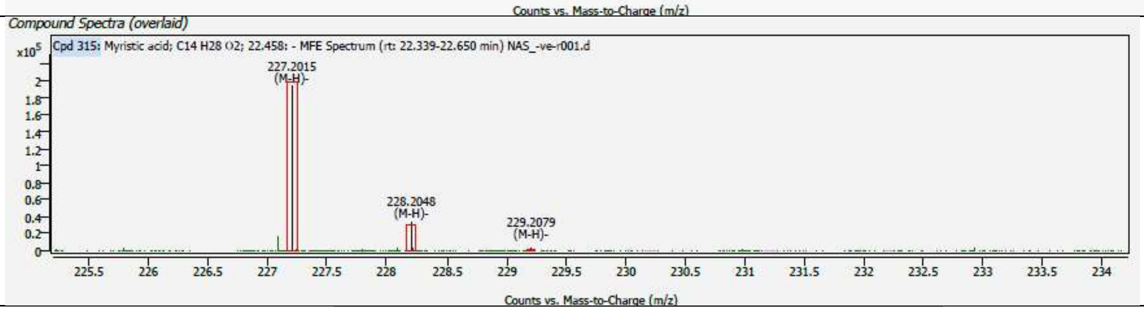
Compound Spectra (overlaid)
x10^5
Cpd 315: Myristic acid; C14 H28 O2; 22.458: - MFE Spectrum (rt: 22.339-22.650 min) NAS_-ve-r001.d
227.2015 (M-H)-
228.2048 (M-H)-
229.2079 (M-H)-
Counts vs. Mass-to-Charge (m/z)

72

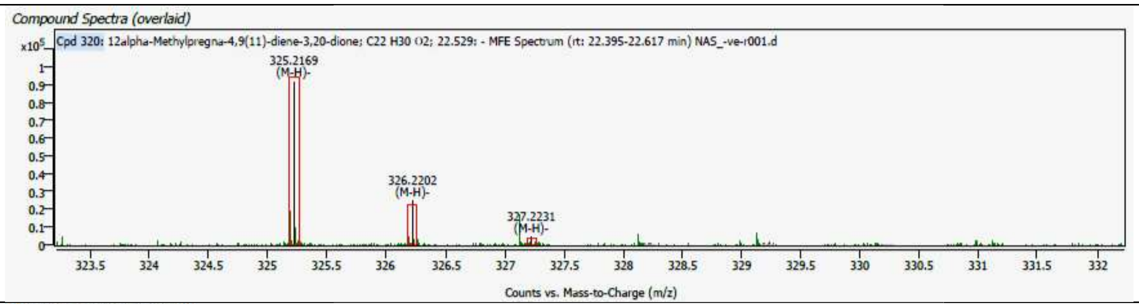

Compound Spectra (overlaid)
Cpd 320: 12alpha-Methylpregna-4,9(11)-diene-3,20-dione; C22 H30 O2; 22.529: - MFE Spectrum (rt: 22.395-22.617 min) NAS_-ve-r001.d
325.2169 (M-H)-
326.2202 (M-H)-
327.2231 (M-H)-
Counts vs. Mass-to-Charge (m/z)

73

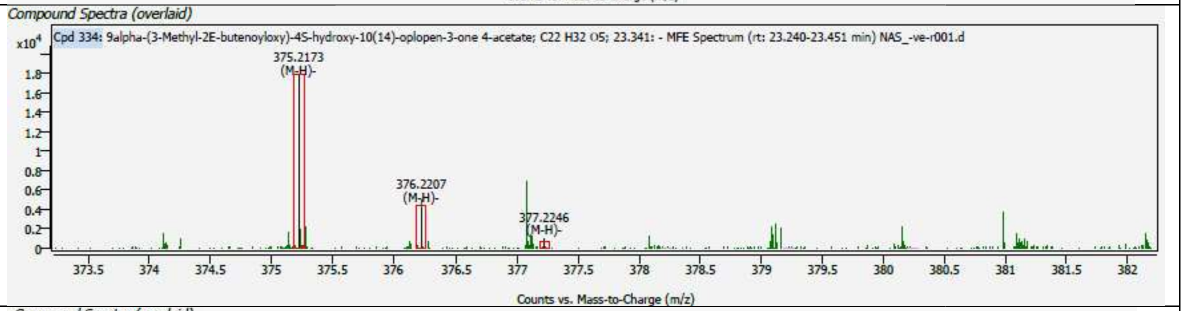

Compound Spectra (overlaid)
Cpd 334: 9alpha-(3-Methyl-2E-butenoyloxy)-4S-hydroxy-10(14)-oplopen-3-one 4-acetate; C22 H32 O5; 23.341: - MFE Spectrum (rt: 23.240-23.451 min) NAS_-ve-r001.d
375.2173 (M-H)-
376.2207 (M-H)-
377.2246 (M-H)-
Counts vs. Mass-to-Charge (m/z)

74

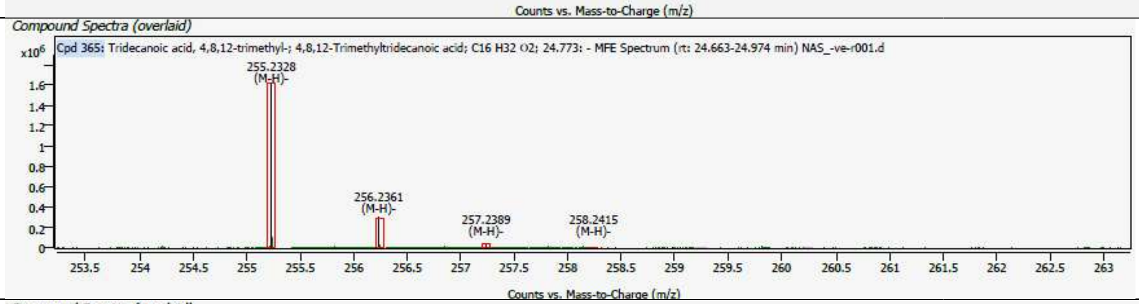

Compound Spectra (overlaid)
Cpd 365: Tridecanoic acid, 4,8,12-trimethyl-; 4,8,12-Trimethyltridecanoic acid; C16 H32 O2; 24.773: - MFE Spectrum (rt: 24.663-24.974 min) NAS_-ve-r001.d
255.2328 (M-H)-
256.2361 (M-H)-
257.2389 (M-H)-
258.2415 (M-H)-
Counts vs. Mass-to-Charge (m/z)

75

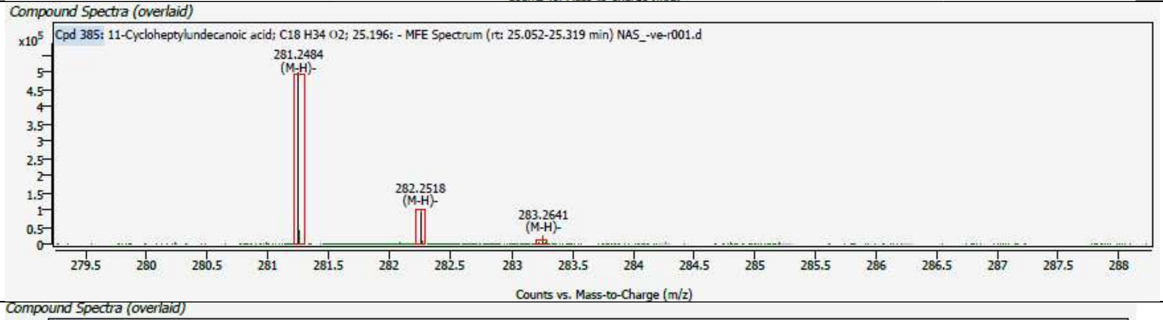

Compound Spectra (overlaid)
Cpd 385: 11-Cycloheptylundecanoic acid; C18 H34 O2; 25.196: - MFE Spectrum (rt: 25.052-25.319 min) NAS_-ve-r001.d
281.2484 (M-H)-
282.2518 (M-H)-
283.2641 (M-H)-
Counts vs. Mass-to-Charge (m/z)

76

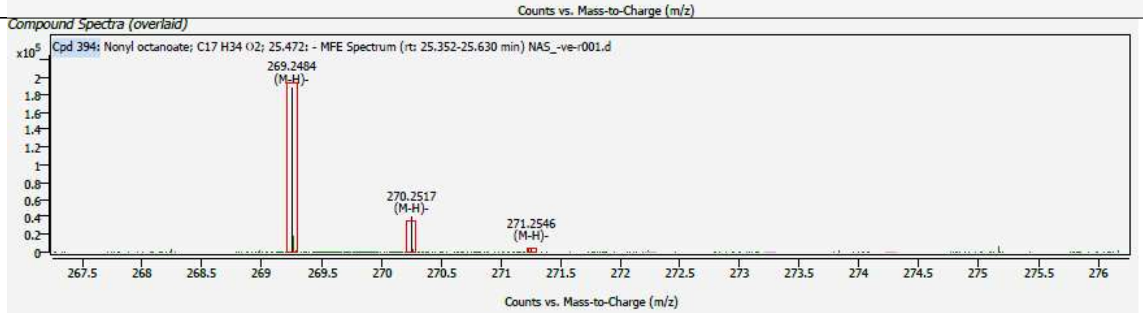

Compound Spectra (overlaid)
Cpd 394: Nonyl octanoate; C17 H34 O2; 25.472: - MFE Spectrum (rt: 25.352-25.630 min) NAS_-ve-r001.d
269.2484 (M-H)-
270.2517 (M-H)-
271.2546 (M-H)-
Counts vs. Mass-to-Charge (m/z)

77	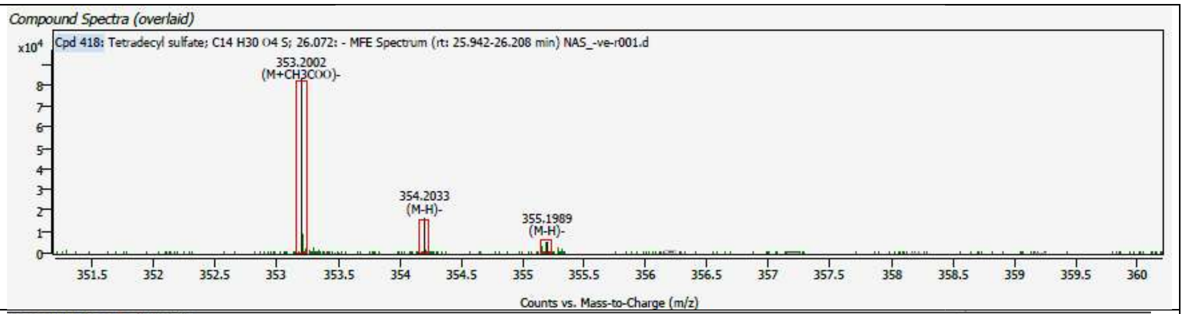Compound Spectra (overlaid)<br>Cpd 418: Tetradecyl sulfate; C14 H30 O4 S; 26.072: - MFE Spectrum (rt: 25.942-26.208 min) NAS_-ve-r001.d<br>x10^4<br>353.2002<br>(M+CH3COO)-<br>354.2033<br>(M-H)-<br>355.1989<br>(M-H)-<br>Counts vs. Mass-to-Charge (m/z)
78	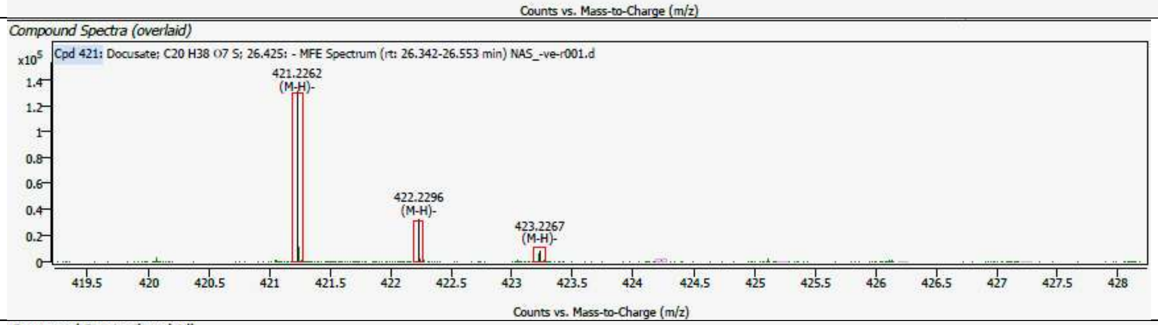Compound Spectra (overlaid)<br>Cpd 421: Docusate; C20 H38 O7 S; 26.425: - MFE Spectrum (rt: 26.342-26.553 min) NAS_-ve-r001.d<br>x10^5<br>421.2262<br>(M-H)-<br>422.2296<br>(M-H)-<br>423.2267<br>(M-H)-<br>Counts vs. Mass-to-Charge (m/z)
79	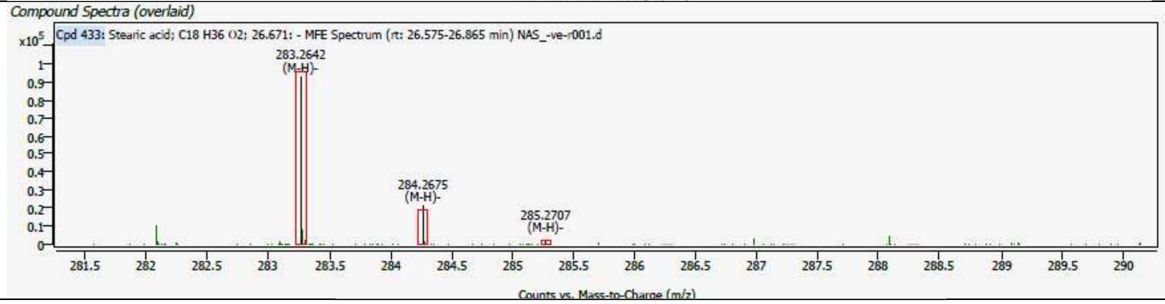Compound Spectra (overlaid)<br>Cpd 433: Stearic acid; C18 H36 O2; 26.671: - MFE Spectrum (rt: 26.575-26.865 min) NAS_-ve-r001.d<br>x10^5<br>283.2642<br>(M-H)-<br>284.2675<br>(M-H)-<br>285.2707<br>(M-H)-<br>Counts vs. Mass-to-Charge (m/z)

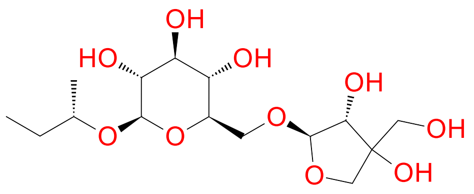

title: PJ_01.sdf

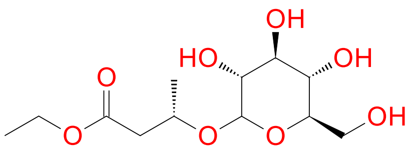

title: PJ_02.sdf

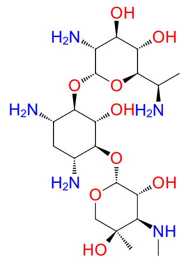

title: PJ_03.sdf

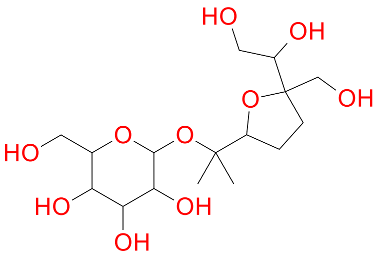

title: PJ_04.sdf

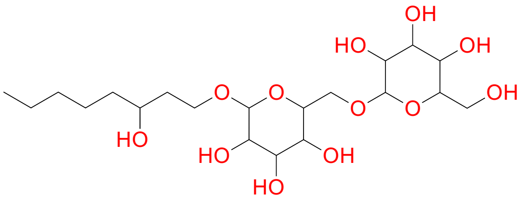

title: PJ_05.sdf

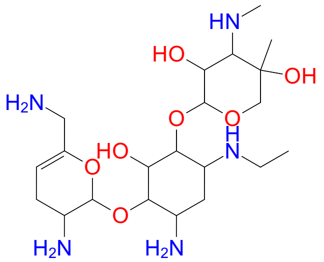

title: PJ_06.sdf

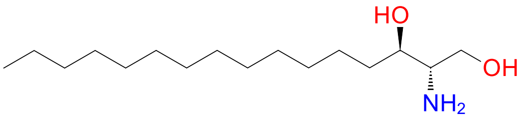

title: PJ_07.sdf

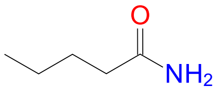

title: PJ_08.sdf

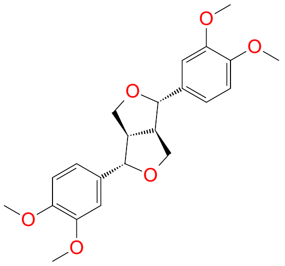

title: PJ_09.sdf

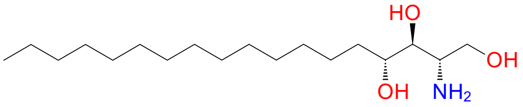

title: PJ_10.sdf

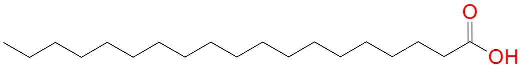

title: PJ_11.sdf

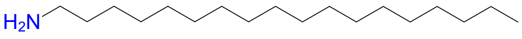

title: PJ_12.sdf

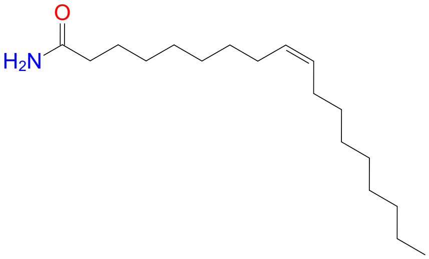

title: PJ_13.sdf

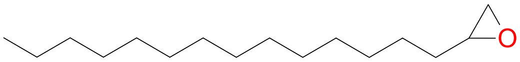

title: PJ_14.sdf

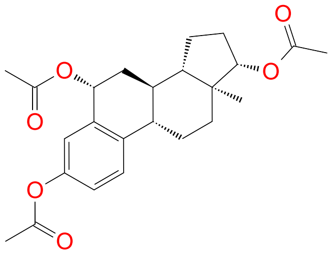

title: PJ_15.sdf

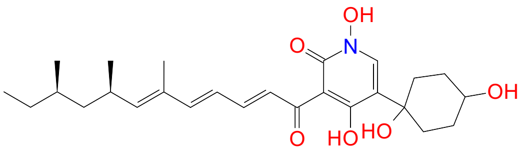

title: PJ_16.sdf

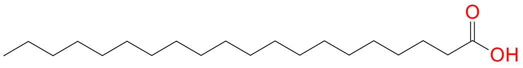

title: PJ_17.sdf

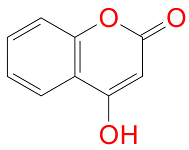

title: PJ_18.sdf

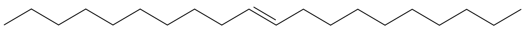

title: PJ_19.sdf

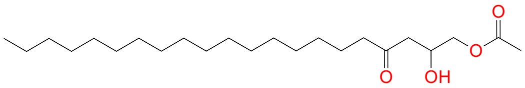

title: PJ_20.sdf

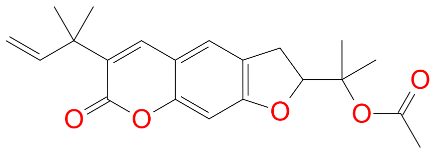

title: PJ_21.sdf

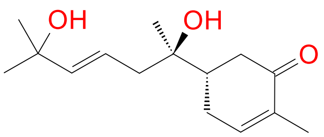

title: PJ_22.sdf

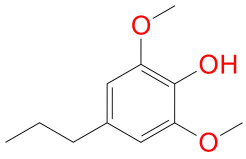

title: PJ_23.sdf

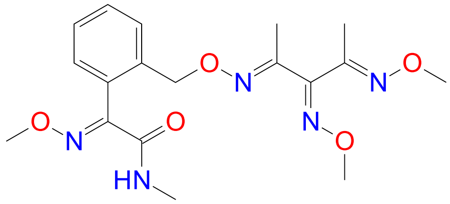

title: PJ_24.sdf

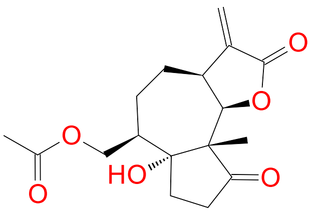

title: PJ_25.sdf

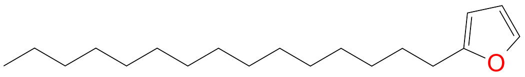

title: PJ_26.sdf

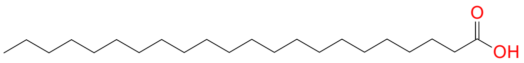

title: PJ_27.sdf

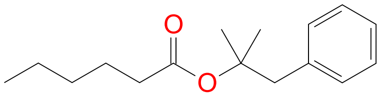

title: PJ_28.sdf

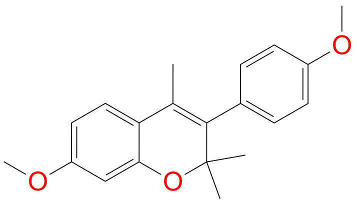

title: PJ_29.sdf

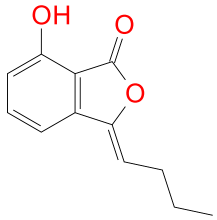

title: PJ_30.sdf

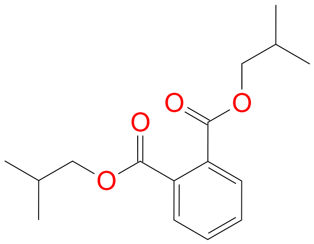

title: PJ_31.sdf

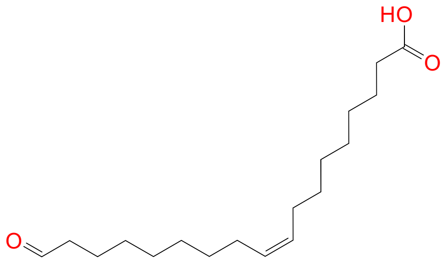

title: PJ_32.sdf

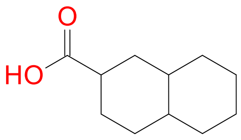

title: PJ_33.sdf

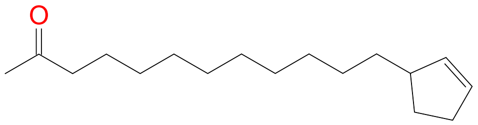

title: PJ_34.sdf

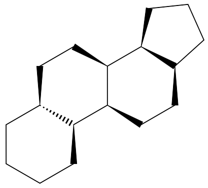

title: PJ_35.sdf

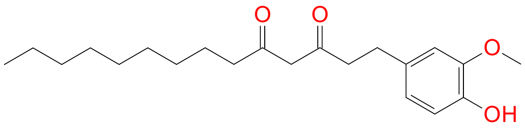

title: PJ_36.sdf

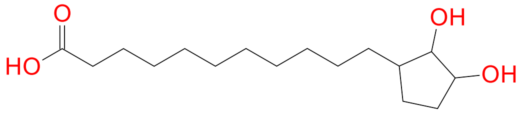

title: PJ_37.sdf

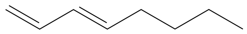

title: PJ_38.sdf

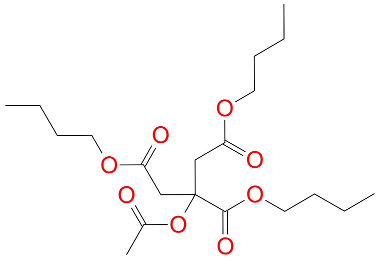

title: PJ_39.sdf

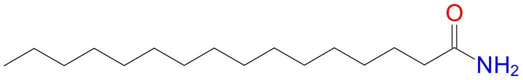

title: PJ_40.sdf

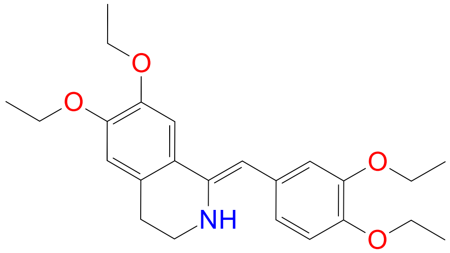

title: PJ_41.sdf

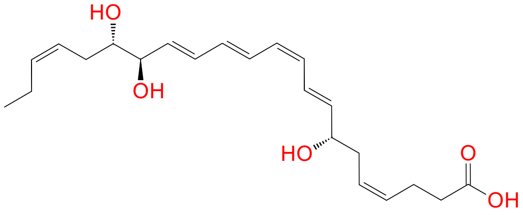

title: PJ_42.sdf

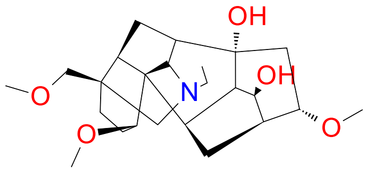

title: PJ_43.sdf

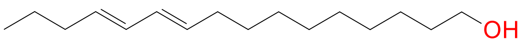

title: PJ_44.sdf

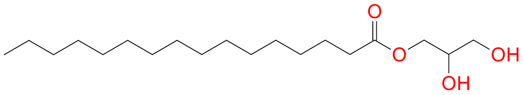

title: PJ_45.sdf

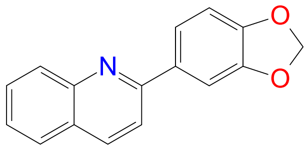

title: PJ_46.sdf

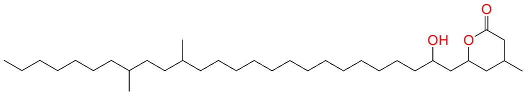

title: PJ_47.sdf

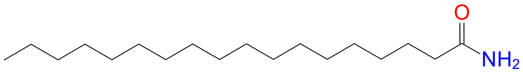

title: PJ_48.sdf

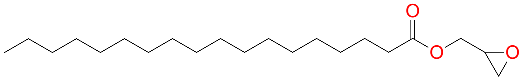

title: PJ_49.sdf

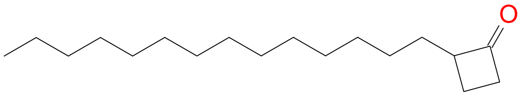

title: PJ_50.sdf

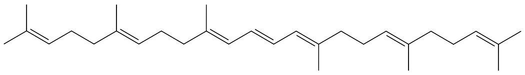

title: PJ_51.sdf

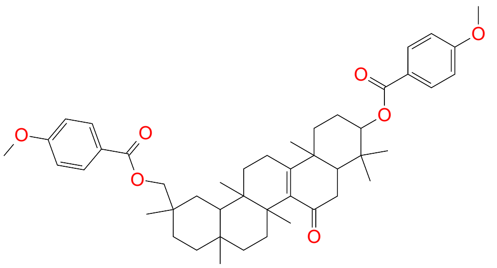

title: PJ_52.sdf

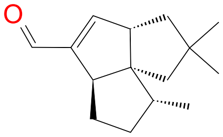

title: PJ_53.sdf

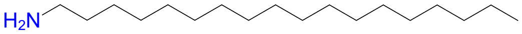

title: PJ_54.sdf

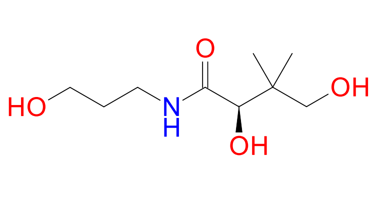

title: PJ_55.sdf

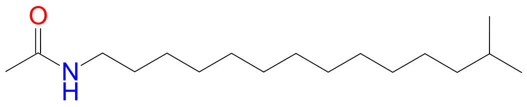

title: PJ_56.sdf

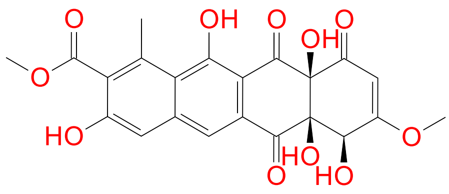

title: PJ_57.sdf

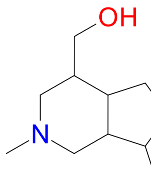

title: PJ_58.sdf

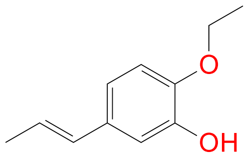

title: PJ_59.sdf

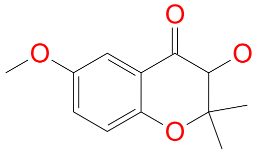

title: PJ_60.sdf

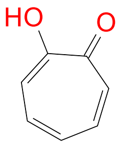

title: PJ_61.sdf

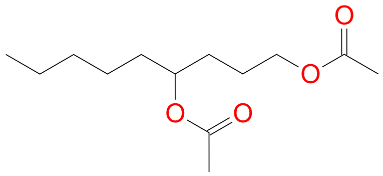

title: PJ_62.sdf

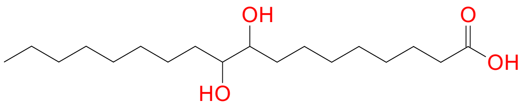

title: PJ_63.sdf

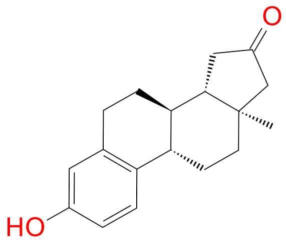

title: PJ_64.sdf

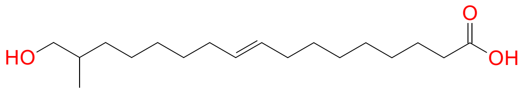

title: PJ_65.sdf

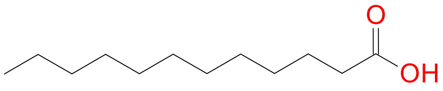

title: PJ_66.sdf

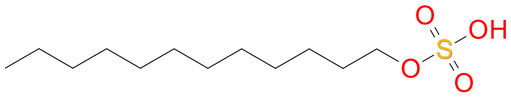

title: PJ_67.sdf

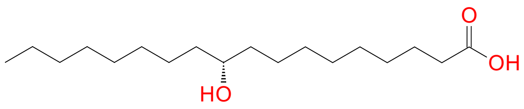

title: PJ_68.sdf

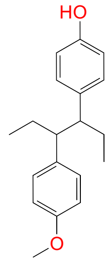

title: PJ_69.sdf

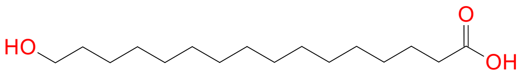

title: PJ_70.sdf

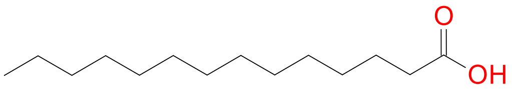

title: PJ_71.sdf

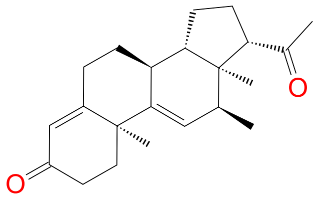

title: PJ_72.sdf

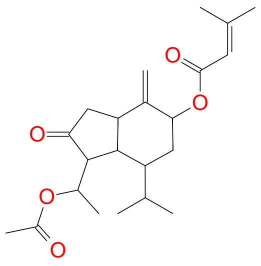

title: PJ_73.sdf

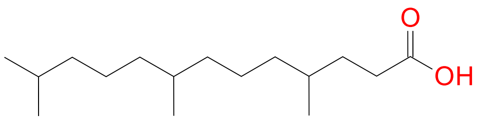

title: PJ_74.sdf

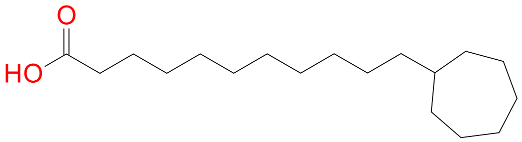

title: PJ_75.sdf

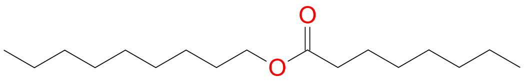

title: PJ_76.sdf

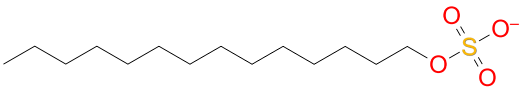

title: PJ_77.sdf

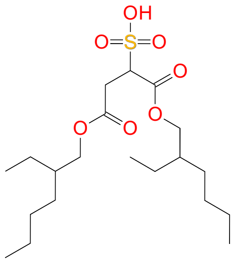

title: PJ_78.sdf

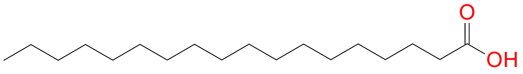

title: PJ_79.sdf

Figure S3. Structure of Compounds identified by UPLC–HRMS both positive and negative mode of ionization
